# The role of ubiquitination in health and disease

**DOI:** 10.1002/mco2.736

**Published:** 2024-09-25

**Authors:** Yan Liao, Wangzheqi Zhang, Yang Liu, Chenglong Zhu, Zui Zou

**Affiliations:** ^1^ Faculty of Anesthesiology Changhai Hospital Naval Medical University Shanghai China; ^2^ School of Anesthesiology Naval Medical University Shanghai China

**Keywords:** protein degradation, protein homeostasis, ubiquitin, ubiquitination

## Abstract

Ubiquitination is an enzymatic process characterized by the covalent attachment of ubiquitin to target proteins, thereby modulating their degradation, transportation, and signal transduction. By precisely regulating protein quality and quantity, ubiquitination is essential for maintaining protein homeostasis, DNA repair, cell cycle regulation, and immune responses. Nevertheless, the diversity of ubiquitin enzymes and their extensive involvement in numerous biological processes contribute to the complexity and variety of diseases resulting from their dysregulation. The ubiquitination process relies on a sophisticated enzymatic system, ubiquitin domains, and ubiquitin receptors, which collectively impart versatility to the ubiquitination pathway. The widespread presence of ubiquitin highlights its potential to induce pathological conditions. Ubiquitinated proteins are predominantly degraded through the proteasomal system, which also plays a key role in regulating protein localization and transport, as well as involvement in inflammatory pathways. This review systematically delineates the roles of ubiquitination in maintaining protein homeostasis, DNA repair, genomic stability, cell cycle regulation, cellular proliferation, and immune and inflammatory responses. Furthermore, the mechanisms by which ubiquitination is implicated in various pathologies, alongside current modulators of ubiquitination are discussed. Enhancing our comprehension of ubiquitination aims to provide novel insights into diseases involving ubiquitination and to propose innovative therapeutic strategies for clinical conditions.

## Graphical abstract

Ubiquitination regulates protein degradation, trafficking, and signaling through a complex machinery involving ubiquitin and various ligases. It is crucial for protein homeostasis, DNA repair, cell cycle regulation, and immune responses. Dysregulation leads to diseases like cancer and neurodegenerative disorders. Therapeutic strategies targeting ubiquitination show promise, and future research aims to explore its emerging roles and technological advancements.

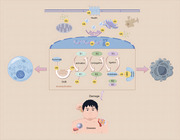

## 1 INTRODUCTION

In the early 21st century, the Nobel Prize in Physiology or Medicine was awarded multiple times to scientists investigating endogenous proteins. Notably, in 2004, Aaron Ciechanover, Avram Hershko, and Irwin Rose were honored with the Nobel Prize in Chemistry for their discovery of the ubiquitin (Ub)‐mediated protein degradation mechanism.^1^ Although the complexities of protein degradation remained elusive for a long time, research from the mid‐1950s to the late 1970s gradually uncovered the role of nonlysosomal pathways under specific physiological conditions, ultimately confirming the Ub–proteasome pathway. This pathway indicates that Ub tagging can regulate a wide range of cellular processes.^2^

**FIGURE 1:**
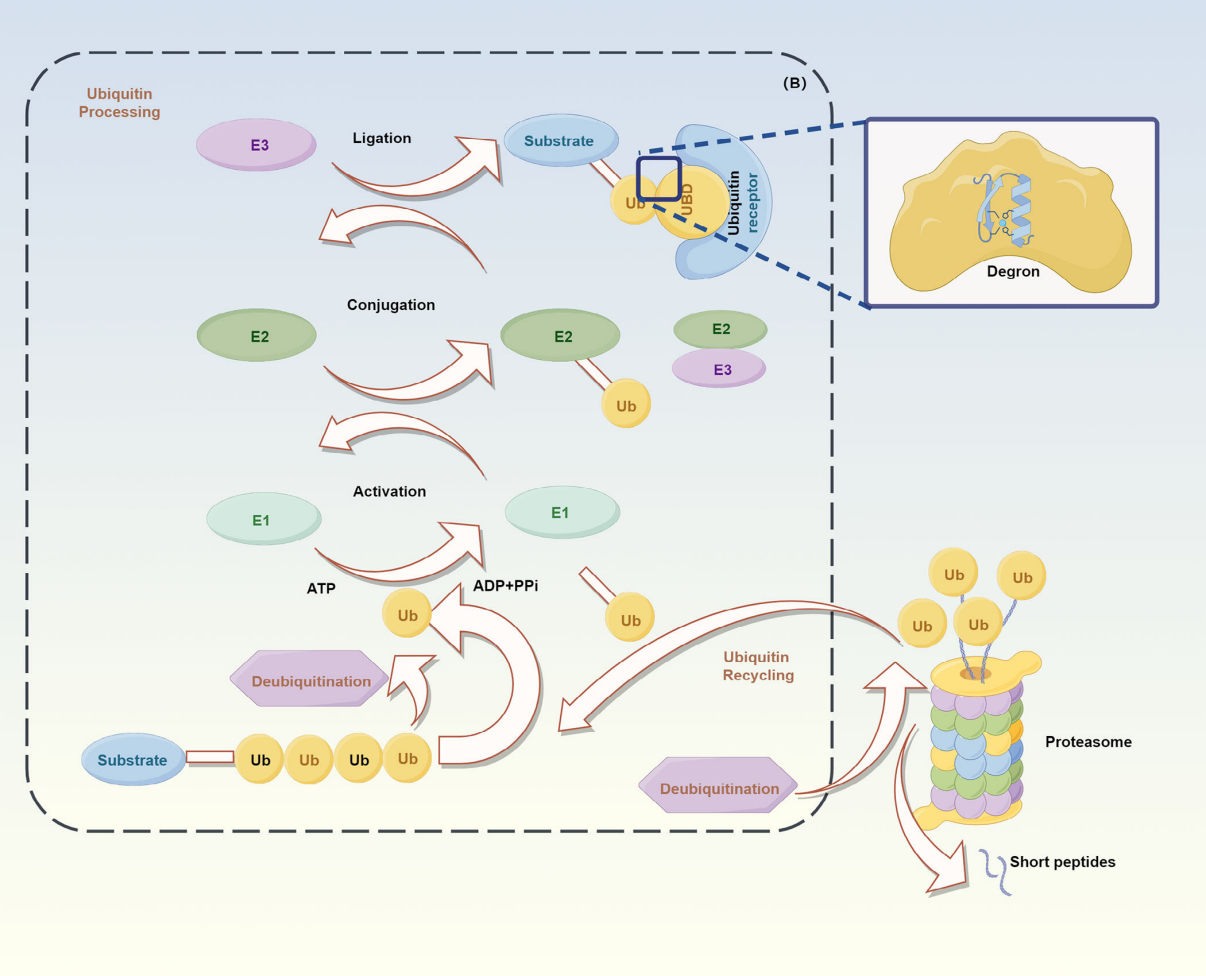
Overview of the ubiquitination process (by Figdraw). Ubiquitination is an enzyme‐catalyzed reaction that plays a crucial role in various biological processes. Substrates are tagged with ubiquitin and transported to the proteasome for degradation, while ubiquitin molecules are recycled by deubiquitinating enzymes.

Research on ubiquitination has significantly advanced our understanding of cellular physiology and various diseases, contributing to the conceptualization of “protein quality control.” Protein quality control refers to the timely elimination of misfolded or damaged proteins to maintain cellular homeostasis. This process is achieved through targeted protein degradation, often involving molecular chaperones. When molecular chaperones fail to repair a protein, degradation becomes the self‐protective choice of cells. Posttranslational modifications (PTMs) are crucial for controlling protein quality and quantity, with common modifications including phosphorylation, methylation, oxidation, nitration, and ubiquitination, which exponentially expand the proteome.^3^ Among these, ubiquitination is the most prevalent PTM involved in protein quality control. The ubiquitination process involves a sequential reaction catalyzed by three enzymes (E1, E2, E3), serving as a regulatory mechanism for numerous cellular activities^4^ (Figure 1). By forming conjugates with diverse topologies, ubiquitination can influence the stability, interactions, localization, or activity of thousands of proteins, thereby providing specific signals for broad cellular control. Ub modification of proteins is a critical determinant of cellular fate and function, playing a pivotal role in maintaining human health and cellular homeostasis. Aberrant ubiquitination frequently leads to disease.^5^

Ubiquitination participates in numerous cellular functions, maintaining homeostasis. However, its dysregulation can cause pathological states, including inflammation, or even cell death. Hence, maintaining normal ubiquitination function is crucial for cellular operations, and targeting ubiquitination offers promising avenues for disease treatment. Here, we focus on the pivotal roles of ubiquitination in health and disease. The Ub system comprises multiple components, each collaborating to target a broad array of substrates, regulating diverse cellular processes and functions. While dysregulation of ubiquitination can lead to disease, this very characteristic make each step of ubiquitination process a potential therapeutic target, providing advanced strategies and directions for disease treatment.

## 2 MECHANISMS OF UBIQUITINATION

### 2.1 Ub and its structure

Ub was first discovered in bovine thymus during the isolation of thymopoietin, a thymic peptide hormone, and was named for its physiological functions, which were not yet fully understood at the time. Initial amino acid sequencing revealed that Ub is a single polypeptide chain containing 74 amino acids.^6^ Subsequently, Wilkinson and Audhya^7^ identified a COOH‐terminal sequence of –Arg–Gly–Gly in the active form of Ub, a 76‐amino‐acid protein that is highly conserved across species, from yeast to plants and mammals. Remarkably, only three positions in the Ub structure differ among mammals, yeast, and plants. Structurally, Ub adopts a compact globular fold known as the “ubiquitin fold” or “ubiquitin superfold,” characterized by a helical five‐stranded sheet at the top and an exposed C‐terminal tail that extends to participate in covalent attachment to target proteins.^8^ Ub is produced either from the fusion of ribosomal proteins (encoded by UBA52 and RPS27A) or through the action of deubiquitinases on polyubiquitin chains to release free Ub (encoded by UBB and UBC).^9^, ^10^ As research deepens, the methods currently used for the purification of Ub protein have been refined and generally fall into three categories: epitope tagging, purification through Ub‐binding domains (UBDs), and the use of antibodies. One of the most commonly used antiUb antibodies is the monoclonal antibody FK2.^11^

Ub contains seven lysine residues at positions Lys6, Lys11, Lys27, Lys29, Lys33, Lys48, and Lys63, along with its amino‐terminal, providing eight potential sites for the molecule to form polymeric chains.^12^, ^13^ Depending on the variability of the lysine linkage sites, Ub chains can be categorized into mono‐ubiquitination or poly‐ubiquitination, with poly‐ubiquitination further subdivided into homogeneous or heterogenous types based on the uniformity of the lysine sites.^14^ Protein ubiquitination occurs in two primary forms, each extensively involved in various cellular processes, such as the intricate regulation of inflammatory signaling pathways,^15^ modulation of cell death,^16^ and control of cell proliferation.^17^ Proteins marked by mono‐ubiquitination typically do not undergo degradation; instead, they often regulate protein function and subcellular localization.^18^ In contrast, polyubiquitinated proteins are predominantly targeted for degradation via the proteasome, thereby participating in more complex signaling events essential for cellular activities.^19^, ^20^ Ub chains linked via K48 predominantly target misfolded or aged proteins for degradation through the proteasomal pathway and regulate the turnover of signaling proteins to constrain various immune signaling cascades.^21^ In contrast, Ub chains linked via K63, while other Ub chains engage in proteasome‐mediated protein degradation, are predominantly found to participate in cellular signaling processes.^22^ Among the various lysine‐linked Ub chains, K33 polyubiquitination is noteworthy for promoting T cell receptor (TCR) ubiquitination and restricting TCR signaling.^23^ Lys11‐linked ubiquitination is often found in mixed or branched chains with Lys48 and Lys63 and facilitate proteasomal degradation. For instance, the anaphase‐promoting complex/cyclosome (APC/C) assembles mixed chains containing Lys11, Lys48, and Lys63 bonds through a two‐step mechanism.^24^ Lys29 linkage is the most abundant atypical linkage in nonstressed cells and has been predominantly studied in yeast. For example, in the Ub‐fusion degradation (UFD) pathway, Lys29 linkage is formed through the concerted action of homologous to the E6‐associated protein C‐terminal (HECT) ligases Ufd4 and Ubr1, playing a role in cellular protein deposition.^25^, ^26^

The K27‐linked Ub chain regulates the NF‐κB subunit IKKγ, playing a role in various diseases. For instance, the pathogen *Shigella* can intercept this Ub chain to suppress immune defense mechanisms of host. The linear Ub chain assembly complex (LUBAC) is a unique structure where one bond is formed through a lysine residue, while the rest of the chain is constructed via the N‐terminal amino group of Ub. LUBAC is composed of SHARPIN, HOIL‐1 (also known as RBCK1), and HOIP. Similarly, linear Ub chains are involved in the activation of the NF‐κB pathway.^27^ In the ubiquitination process, in addition to the linkage through lysine residues on Ub itself, Ub can also form covalent bonds via peptide linkage with the N‐terminal α‐amino group, resulting in the formation of linear polyubiquitin chains or N‐terminal Ub fusions.^28^, ^29^

**FIGURE 2:**
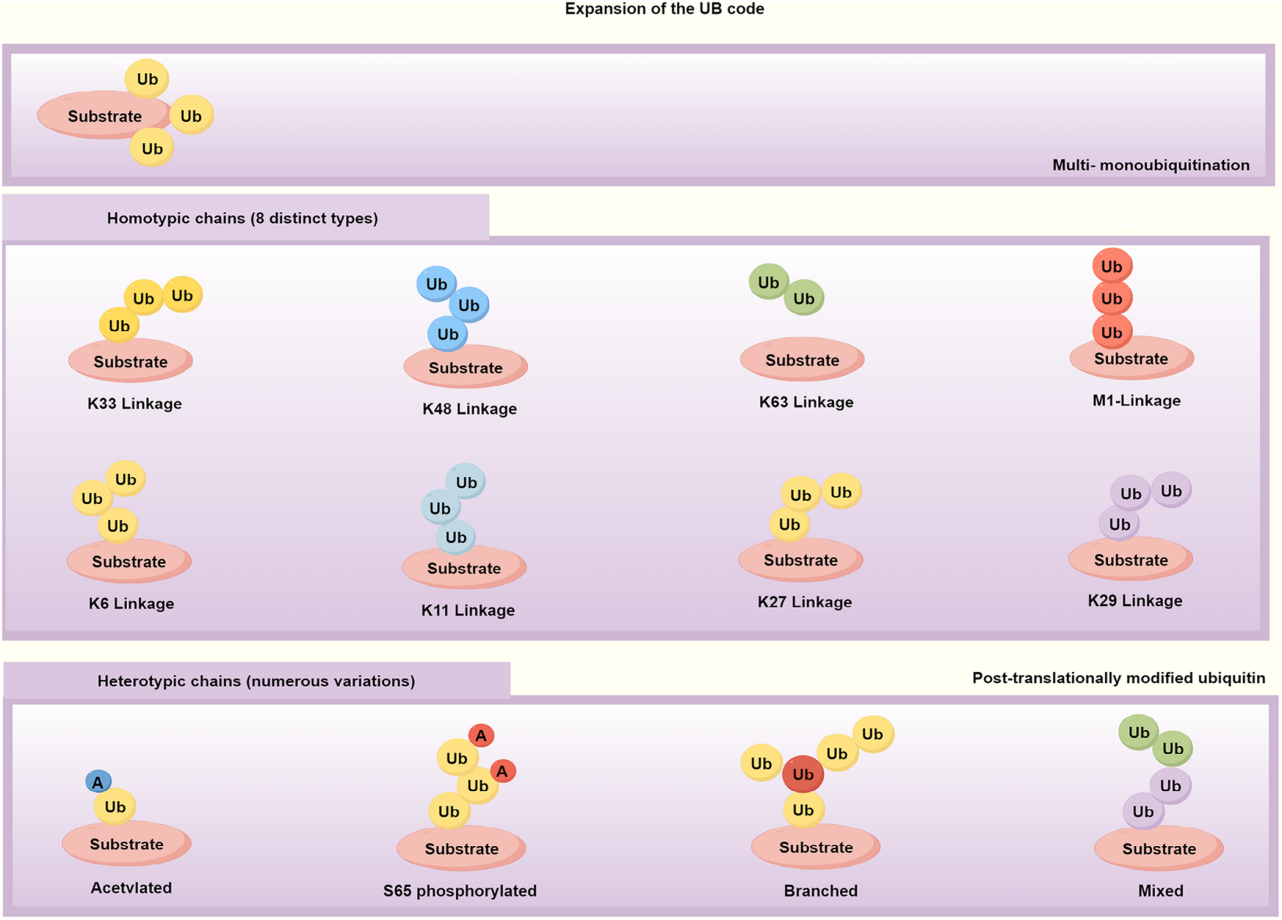
Overview of the ubiquitin chain types (by Figdraw). Different types of ubiquitin chains: monoubiquitination and polyubiquitination. Polyubiquitin chains can be homotypic or heterotypic, indicating the uniformity or diversity in the linkage types within the chain.

Ubiquitination is inherently a PTM process; however, Ub itself is a protein, and its seven lysine residues can undergo further modifications, such as phosphorylation, acetylation, and phosphoribosylation. Under the influence of electrostatic and spatial effects, the acetylation of different lysine residues in Ub molecules leads to mono‐acetylated Ub monomers that exert specific impacts on Ub structure, with different acetylated variants connecting to distinct cellular pathways.^30^, ^31^ The pathogenic *Legionella pneumophila* effector protein SdeA has been demonstrated to mediate NAD‐dependent and ATP‐independent transfer of Ub to host proteins. Ub phosphoribosylation prevents the activation of conventional ubiquitination cascade enzymes E1 and E2, thereby regulating various cellular activities.^32^ This renders the ubiquitination modification process even more intriguing and unpredictable (Figure 2).

### 2.2 Enzymes involved in ubiquitination: E1, E2, and E3 ligases

The human genome encodes two E1 enzymes, at least 38 E2 enzymes, and at least 600 E3 enzymes.^33^ Generally, enzyme‐mediated ubiquitination can be divided into two processes: the enzymatic cascade and the reversible process.^11^ Ub activation involves the formation of a thioester bond between the E1 enzyme and the Ub molecule. The activated Ub is then transferred to an E2 Ub‐conjugating enzyme, which interacts with an E3 Ub ligase to facilitate the transfer of Ub to the lysine residue on the substrate protein. In the presence of ATP, E1 enzyme adenylates the C‐terminus of Ub, then transfers Ub to a conserved Cys residue in E1, resulting in the formation of E1‐Ub thioester, along with the release of AMP and pyrophosphate.^10^ The ultimate outcome of the ubiquitination cascade is the linkage of the ε‐amino group of the substrate's lysine side chain to the C‐terminus of Ub.^34^

The E1 enzyme is a monomeric protein with a molecular weight of 110−120 kDa, composed of four structural modules: active adenylation domain, first catalytic cysteine half‐domain (FCCH), second catalytic cysteine half‐domain (SCCH), and ubiquitin fold domain (UFD).^35^ The catalytic cysteine half‐domains, FCCH and SCCH, include the E1 active site cysteine, which is inserted into each adenylation domain.^36^ These domains are crucial for the enzyme's function. Furthermore, the structure includes a four‐helix bundle, which represents a secondary insertion in the inactive adenylation domain immediately following FCCH; and finally, the C‐terminal UFD recruits specific E2, which is essential for subsequent steps in the ubiquitination process.^35^, ^37^ In all eukaryotes, E1 contains a duplicated sequence of domains derived from bacterial MoeB and ThiF proteins.^38^ As a multidomain enzyme, each domain of E1 plays distinct roles in its three catalytic activities: adenylation, thioester bond formation, and thioesterification. The E1 enzyme is regarded as the gatekeeper of the Ub signaling pathway, as it catalyzes the activation of Ub and its transfer to dozens of homologous E2‐conjugating enzymes in a process called E1–E2 thioesterification.^37^ During the ubiquitination process, E1 alters the ubiquitination pathway through conformational changes. Ub thioester (Ub(t)) interacts with E1 in an open conformation, while Ub(t) interacts with E2 in a completely different closed conformation, representing two states before and after thiol ester transfer. The SCCH domain can bind two ubiquitins, acting as a molecular switch. During this process, the SCCH domain undergoes a 106° rotation, bringing the catalytic cysteine closer to the Ub C‐terminus.^39^

The UBE1‐E2 (Ubc4)/Ub/ATP–Mg complex represents the crystallographic structure generated during the binding of E1 and E2, stabilized by a disulfide bond between their active sites. This structure reveals the combinatorial recognition by the E1 UFD and the Cys domain of E2, where conformational changes in E1 lead to the convergence of the active sites of E1 and E2 during thioester transfer. In this process, E2 binds to the Cys domain of E1 in a fully open configuration within the Ub–E1–E2 complex.^40^ The E2 enzyme plays a pivotal role in determining the length and linkage type of the Ub chain.^41^ All E2 enzymes possess a core catalytic domain of approximately 150 amino acids, known as the UBC domain, typically comprising four α‐helices and a four‐strand β‐sheet. Some E2s featuring short N‐ and/or C‐terminal extensions that facilitate essential E2 functions. E2s primarily exist in the form of E2–Ub conjugates, being prepared for reaction. In the absence of an E3 ligase, the E2–Ub conjugate exhibits a low rate of Ub transfer, thereby minimizing energy loss.^42^ Each E2 enzyme interacts with one E1 enzyme and one or more E3 enzymes. Additionally, E2s may directly bind to target proteins, helping to determine the site and manner of Ub modification on the target protein.^43^ Moreover, beyond their role in the ubiquitination cascade, E2 Ub‐conjugating enzymes can regulate the function of other enzymes independently. For example, the E2–Ub conjugate acts as a novel regulator of the OspG effector kinase function in eukaryotic host cells, where OspG is a Shigella effector kinase.^44^, ^45^ E2‐conjugating enzymes can also enhance the activity of deubiquitinating enzymes (DUBs) like OTUB1 through induced conformational changes.^46^

E3 ligases, despite their diversity, are constructed from a small number of basic catalytic cores with various substrate recruitment modules and regulatory elements, allowing them to process different protein substrates and respond to diverse cellular signals.^47^ E3 ligases are classified into three main categories: really interesting new gene (RING) ligases, HECT ligases, and RING‐in‐between‐RING (RBR) Ub ligases. RBR ligase contain three tandemly arranged zinc‐binding domains that mediate the direct transfer of Ub from E2 enzymes to target proteins.^48^, ^49^ Throughout the ubiquitination process, E1 and E2 are not always indispensable. Certain members of the Parabacteroides distasonis family, which interact with multiple Rab small GTPases associated with the endoplasmic reticulum (ER), can catalyze ubiquitination without E1 and E2 enzymes.^50^ When E2 and E3 enzymes are linked, E1 is mutually exclusive, meaning that E1 must dissociate from E2 before E2 binding to E3. This is due to the specific residues on the N‐terminal helix of E2 UbcH7, which interact with both the HECT domain‐containing E3 ligase E6AP1 and the RING domain‐containing c‐Cbl2, while also being capable of interacting with the APPBP1–UBA3 of E1.^51^ RING E3 ligases contain a RING (or RING‐like) domain responsible for binding to E2 and stimulating Ub transfer, with two conserved Zn^2+^ cross‐linking the structure.^52^ The RING domain binds to the N‐terminal helix of the E2 conjugating enzyme, and it binds even more tightly to the E2–Ub conjugate. In contrast to RING E3 ligases, HECT domain E3 ligases catalyze two distinct reactions: first, a thioesterification reaction where the active site cysteine of E2 is transferred to a cysteine in the HECT domain, followed by a subsequent reaction where the substrate lysine attacks the HECT‐Ub thioester.^53^ Additionally, RBR proteins represent a unique family of RING‐HECT hybrid E3s, possessing characteristics of both RING and HECT E3 ligases but catalyzing ubiquitination in a unique manner and autoinhibiting their activity, such as the human homolog ariadne (HHARI) and Parkin, whose RING2 structures contain a catalytic cysteine that mediates ubiquitination in a manner similar to HECT, while RING1 recruits charged E2.^54^ The binding of HOIP with HOIL‐1L and SHARPIN in linear Ub chains alleviates this autoinhibition and increases catalytic activity.^55^, ^56^

Beyond the three types of E3 ligases mentioned above, the recently discovered neuronal‐associated MYCBP2/Phr1 represents a new class of RING‐connected E3s with esterification activity and intrinsic selectivity for threonine over serine. MYCBP2 contains two essential catalytic cysteine residues that transfer Ub to the substrate via thioester intermediates, indicating that higher eukaryotes can also undergo nonlysine ubiquitination.^53^ The site where E3 ligases connect with their substrates is referred to as a degron, a specific amino acid sequence of relatively short length (5–20 amino acids) that contains a particular motif for E3 ligases for substrate recruitment. PTMs by kinases and other enzymes on degrons often play a crucial role in determining the timing of E3–substrate interactions and integrating them with upstream events. The presence of degrons ensures a certain degree of precision in the tagging of target proteins.^57^ However, degrons are not essential for substrate recognition, as certain proteins can still bind to E3 enzymes through their native structures.^47^, ^58^

Due to the presence of deubiquitinases (DUBs), ubiquitination is a reversible protein modification. Currently, approximately 100 DUBs have been identified.^59^ DUBs can exhibit internal, external, or global‐type activities by cleaving within the chain, cutting from one end of the chain, or removing the entire chain at once.^60^ Given the diversity and complexity of Ub chains, recognizing various Ub chains by DUBs is a challenging task. The human genome encodes over 70 DUBs, which can be classified into seven types: USPs (Ub‐specific proteases), UCHs (Ub carboxyl‐terminal hydrolases), MJDs (proteases containing the Machado‐Josephin domain), OTUs (ovarian tumor proteases), MINDYs (motifs interacting with Ub‐like new DUB family), ZUP1 (zinc‐finger Ub peptidase), and JAMMs (JAB1, MPN, MOV34 family).^60^ The first six DUB families are cysteine peptidases, while JAMMs are zinc metallopeptidases.^61^ Overall, most human DUBs are thiol‐based proteases, typically possessing a catalytic triad that includes a catalytic cysteine involved in nucleophilic attack, a neighboring basic histidine that lowers the p*K*a of cysteine to enhance its nucleophilicity, and usually a third acidic residue—often aspartate, asparagine, or glutamate—that further polarizes the basic histidine.^60^ In essence, most DUB‐catalyzed reactions involve the proteolytic cleavage of the bond between a lysine ε‐amino group and a carboxyl group at the C‐terminus of Ub. DUBs recognize the R72 residue at the C‐terminal tail of Ub to form an acyl intermediate, which is then hydrolyzed by a water molecule to complete the catalytic cycle.^62^, ^63^ By cleaving Ub attached to the substrates or within the Ub chains, DUBs play a crucial role in regulating fundamental cellular processes, either as switches to remove Ub signals or as rheostats to fine‐tune the amount and type of ubiquitination.^64^ Based on this structural foundation, the physiological functions of DUBs are diverse: acquiring free Ub; stabilizing proteins to prevent degradation; and trimming Ub chains to edit the form of Ub modification.^65^

In summary, the ubiquitination process begins with the formation of a thioester bond between the Cys residue of the E1 enzyme and the C‐terminus of Ub, a process that requires ATP to release energy. Once E1 is activated, Ub is transferred to the Cys residues on over 40 different E2 Ub‐conjugating enzymes. Last, E3 ligases transfer the Ub molecule to a specific substrate.^66^ Additionally, DUBs serve as “recycling stations” during the ubiquitination process.

### 2.3 Ub‐binding proteins and receptors

UBDs are modular elements within proteins that allow for noncovalent binding to Ub, enabling interactions that facilitate mutual regulation.^67^ Due to the reversible nature of ubiquitination, the binding between UBDs and Ub is relatively weak, especially in monoubiquitin chains (with kDa values ranging from 10 to 500 µm). This weak interaction facilitates the assembly and disassembly of Ub and substrates, benefiting various biological processes.^67^ Given that the structure of Ub is highly conserved in organisms, the structural diversity and complexity of UBDs are beneficial to multifunctionality of ubiquitination. The existence of different UBDs adds more possibilities to ubiquitination based on various Ub chain structures. According to their structures, UBDs can be classified into several types, including α‐helices, zinc fingers, pleckstrin homology domains, and Ubc domains present in E2 enzymes, among others.^68^ These domains reside in different proteins and perform various functions. For instance, UBDs containing α‐helical structures often bind to hydrophobic patches on the β‐sheet of Ub molecules.^69^ Similarly, β‐sheets in E2 enzymes, such as the E2 Ub‐conjugating enzyme UBCH5c, can also bind to Ub molecules.^70^ The first characterized Ub‐binding site was identified in the proteasomal subunit S5a/RPN10 protein.^71^ A sequence motif known as the Ub‐interacting motif (UIM) was identified through hidden Markov modeling and iterative database searches based on the S5a sequence, representing a genuine Ub‐binding motif.^72^ Ub‐associated (UBA) domains can directly bind to Ub and are common sequence motifs shared by protein subsets involved in ubiquitination or deubiquitination reactions.^73^ The discovery of UIM and UBA motifs marked the beginning of research into UBDs. Proteins typically contain multiple copies of UBD structures; for example, UIMs often occur in tandem, and such tandem arrangements can exhibit different functions.^72^ A prominent example is seen in proteins involved in various signaling pathways, where UIM‐containing regions bind to Ub, thereby mediating signal transduction. This is seen in proteins such as Eps15, Eps15R, and epsins, which are induced by active tyrosine kinases.^74^ The role of ubiquitination in various signaling pathways will be discussed in detail in the following sections.

In addition to UBDs, proteasomes possess receptors that facilitate the recognition of ubiquitinated proteins, guiding them to the proteasome for degradation. In yeast proteasomes, Rpn10, Rpn13, Rad23, Dsk2, and Ddi1 have been identified as key components that assist in the docking of Ub molecules with the proteasome. In mice, RPN10 and RAD23 are essential, suggesting that they may have more complex and unique roles.^75^ For instance, the two UIMs of RPN10 in yeast (corresponding to S5a in humans) adopt a helical configuration, capable of binding polyubiquitin chains. Due to the separation of the two UIMs by flexible linker regions, they also possess the ability to independently bind monoubiquitin.^76^, ^77^ The yeast protein Rad23, which belongs to a family of proteins containing an N‐terminal Ub‐like (UBL) domain, can bind to the proteasome. Experimental studies using model proteins have shown that when the internal unstructured loop of Rad23 is sufficiently long, it can bind to the proteasome.^78^ Ubiquitinated proteins can also be degraded through the autophagy pathway, where specific receptors recognize and bind Ub. Autophagy receptors such as p62, NBR1, OPTN, and NDP52 can simultaneously bind Ub and the cargo to be degraded, leading to the initiation of autophagy.^79^ For example, p62 contains a UBA domain, and ubiquitination of p62 disrupts the dimerization of the UBA domain, enhancing its ability to selectively recognize polyubiquitinated cargoes for autophagy.^80^ The mechanism of p62 ubiquitination involves acetylation of K420 and K435 in the UBA domain, with acetylation of K435 directly increasing the affinity of the UBA–Ub interaction.^81^ Similar to p62, other autophagy‐related proteins, such as NBR1, OPTN, and NDR52, also contain UBA domains in their structures, granting them the ability to bind Ub.^82^

## 3 REGULATION OF CELLULAR FUNCTIONS BY UBIQUITINATION

### 3.1 Protein degradation via the Ub–proteasome system

In the 1930s, isotope labeling techniques confirmed the lysosome as the principal site for cellular protein degradation, mediated by resident acid‐dependent proteases. However, this view was challenged with evidence that the half‐lives of most cellular proteins were insensitive to lysosomal alkalinization.^83^ Until the discovery of the Ub–proteasome pathway in 1984, the scientific community was perplexed by the fact that ATP was required for the degradation of proteins.^84^ The proteasome pathway primarily degrades short‐lived cytosolic and nuclear proteins as well as misfolded proteins from the ER. Autophagy engulfs large protein aggregates and damaged organelles by forming double‐membraned autophagosomes, which subsequently fuse with lysosomes to degrade the substrates.^85^ The UPS was initially identified from an ATP‐dependent protein hydrolysis system in reticulocytes.^86^ The proteasome, a multisubunit protease now known as the 26S proteasome,^58^ functions as a compartmental protease within the AAA+ (ATPases associated with various cellular activities) protein family. It utilizes ATP hydrolysis to unfold substrates and translocate the denatured polypeptides into an internal degradation chamber for proteolytic cleavage. This capability of unfolding native structures allows the proteasome to regulate the eukaryotic proteome, degrading many regulatory proteins in addition to damaged or misfolded peptides.^87^ The autophagic and proteasome pathways are interconnected. Inhibition of autophagy leads to increased levels of proteasomal substrates. For instance, studies have demonstrated that upon autophagy inhibition, p62 (also known as A170/SQSTM1) accumulates and subsequently inhibits the clearance of ubiquitinated proteins by delaying their delivery to the proteasome.^85^ Studies have shown that the mechanisms by which misfolded proteins from different cellular compartments are transported to the proteasome for degradation vary. For cytoplasmic proteins, degradation requires tagging with mixed Ub chains linked through K48 and K11, followed by interaction with specific chaperone proteins. In contrast, in the nucleus, the proteasomal degradation of misfolded proteins primarily requires K48‐linked Ub chains and recognition by Ub protein Dsk2 for subsequent entry into the proteasome.^88^ The proteasome, formally known as the 26S proteasome, is a complex enzyme composed of a 20S core particle (CP) and 19S regulatory particles (RPs). These two particles work together to regulate protein degradation within the proteasome. The hydrolytic pathway of the proteasome resides within the cavity of the 20S CP, structurally capped at one or both ends by the 19S RPs, thus controlling access to the cavity.^87^ Spatially, the active sites of the CP and RPs are separated but interconnected. In yeast, the proteasome‐associated protein Ecm29 maintains the CP–RP complex, with ATP or ADP being indispensable for the stability of this complex.^89^ Binding of ubiquitinated proteins to the 26S complex, although of high affinity, is reversible and can be disrupted by competition with other UBDs or high salt concentrations. This binding can occur even at low temperatures, such as 4°C. However, tighter binding requires ATP hydrolysis and the presence of loosely folded regions in the substrate protein, as the energy from ATP hydrolysis is converted into mechanical force.^90^ Structural studies, both in vivo and in vitro, have shown that the 26S proteasome exhibits multiple conformations, categorized into the substrate‐free (s1) state and the substrate‐processing (s3‐like) state. The s1 state is the predominant conformation of the ATP‐bound proteasome, whereas the s3‐like state is more conducive to processive degradation.^91^ The interaction of substrates with the AAA+ motor pore loops of the 26S proteasome drives this overall conformational switch, a process requiring intricate coordination. Substrates with short or low‐complexity initiation regions rapidly enter the central channel of the proteasome but may fail to stably engage with the AAA+ pore loops, leading to their swift release and a significant increase in substrate KM. This is mainly due to the increased off‐rate of substrates following initial Ub interaction.^91^ Recent research has revealed that even uncapped 20S proteasomes are capable of cleaving partially unfolded proteins or proteins with disordered regions. This degradation pathway is strictly regulated by a family of catalytic core regulators.^92^ Therefore, structural changes in the proteasome are crucial for its ability to bind target proteins and facilitate their degradation.

### 3.2 Ub‐mediated protein trafficking and localization

**FIGURE 3:**
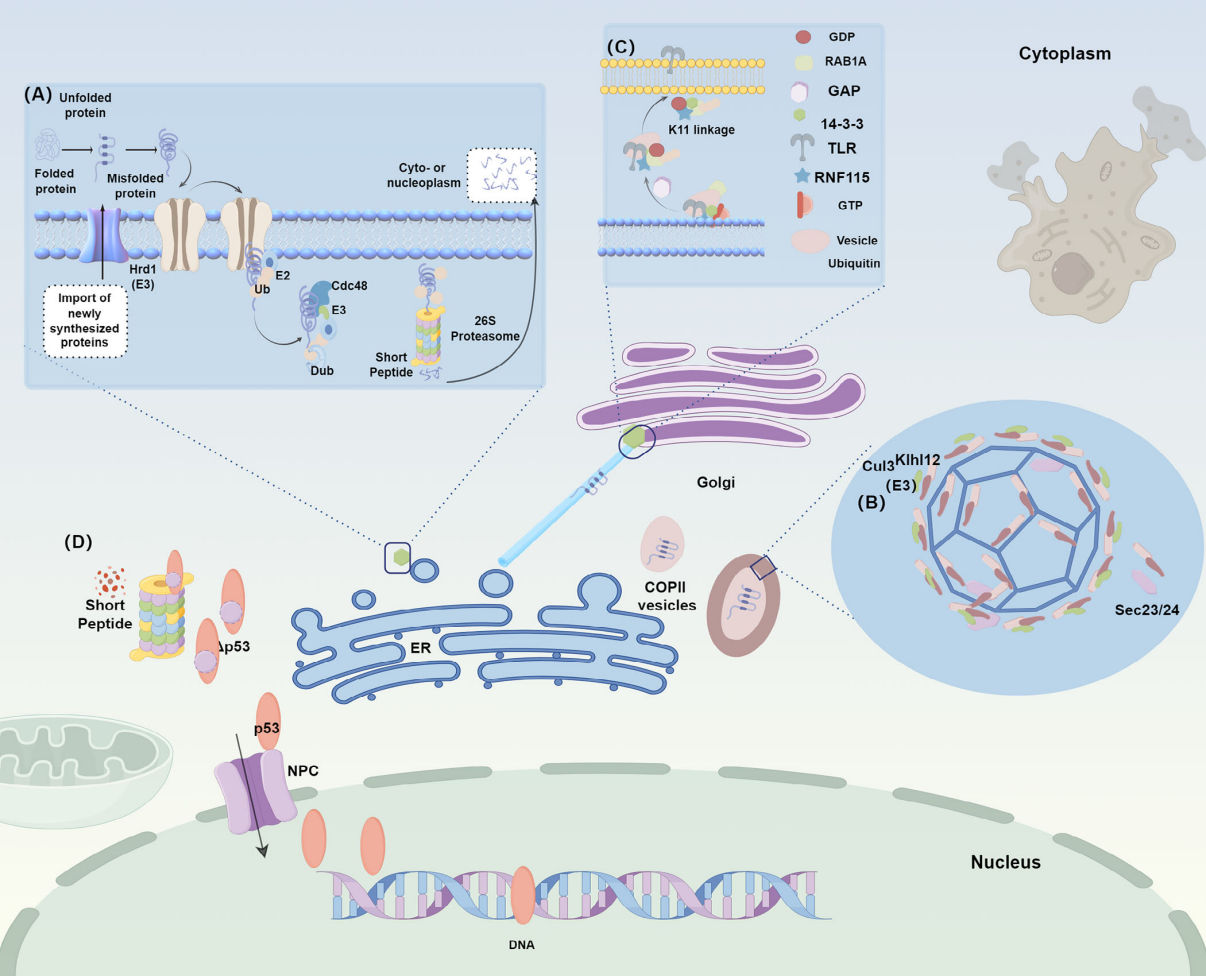
Ubiquitin‐mediated protein trafficking and localization (by Figdraw). (A) Ubiquitin‐mediated protein degradation in the endoplasmic reticulum (ER) involves tagging misfolded proteins for transport to the proteasome. (B) Ubiquitination facilitates the reorganization of COPII vesicles, allowing them to form larger vesicles capable of accommodating substrates. (C) In the Golgi apparatus, ubiquitination mediates the relocation and transport of proteins. (D) Ubiquitin‐mediated transport of proteins within the nucleus ensures proper protein localization and function. Dub, deubiquitinases; NPC, nuclear pore complex; ER, endoplasmic reticulum.

One critical function of ubiquitination is the regulation of protein trafficking and endosomal sorting. One illustrative example of ubiquitination's role in protein trafficking is its involvement in the secretion of secretory proteins. These proteins begin their journey in the ER, where they are folded and assembled into oligomeric complexes. Following a series of modifications, they are packaged into vesicles and transported to the Golgi apparatus.^93^ Misfolded polypeptides, however, can be retrotranslocated back to the cytosol and degraded by the UPS, a process known as ER‐associated degradation (ERAD) (Figure 3A).^94^ The ER contains a variety of autophagy adaptors, such as transmembrane receptors FAM134B and UBX2, and soluble receptors like p62, which can bind ubiquitinated substrates (e.g., TRIM13) and mediate ER‐phagy. In this process, Hrd1, an E3 Ub ligase, facilitates the translocation of ERAD substrates to the cytosol, reorienting them through the ER membrane via the central pore of the Type II AAA‐ATPase Cdc48 (in yeast) or VCP (also known as p97, in mammals), promoting their extraction into the ERAD pathway. Misfolded proteins are subsequently degraded into short peptides and transported into the cytosol or nucleus with the assistance of cytosolic chaperones and transport factors.^95^, ^96^ Furthermore, the unfolded protein response and ERAD interact in a coordinated manner with the UPS and autophagy to mitigate protein misfolding or its consequences.^97^ Upon leaving the ER, the packaged proteins are transported in vesicles or specialized tubules to the Golgi apparatus.^98^ Ubiquitination can increase the size of the export vesicles by approximately fivefold, enabling the accommodation of cargoes, such as collagen, which are 300−400 nm in size, into vesicles with a diameter of only about 60−80 nm. Cul3–Klhl12 is a regulatory factor for COPII coat formation and catalyzes the monoubiquitylation of the COPII‐component SEC31, driving the assembly of large COPII coats^99^ (Figure 3B).

Beyond its established functions, Ub has been identified as a key protein sorting signal, facilitating the transport of damaged and downregulated proteins to the lysosome. This process operates via a unified mechanism in which Ub attachment signals proteins for clathrin‐mediated internalization and endosomal sorting. For instance, studies in mammalian cells have demonstrated that cell surface proteins such as the epidermal growth factor receptor (EGFR) and the epithelial sodium channel directly associate with Ub ligases, supporting the role of Ub as a transport signal within endocytic pathways. Within this model, Ub, as an independent sorting signal, provides a Ub surface for endosomal receptor binding. The underlying mechanisms of Ub‐dependent sorting involve clathrin‐mediated internalization, followed by sorting into multivesicular bodies through the endosomal sorting complex required for transport (ESCRT) system.^100^

Secretory proteins undergo further packaging and sorting within the Golgi apparatus. Ubiquitination plays a pivotal role in this process as a sorting signal, facilitating the binding of Ub to receptors on the Golgi membrane and the accumulation of proteins in clathrin‐coated vesicles. In yeast, proteins such as Gga1 and Gga2 (corresponding to human GGA1, 2, 3) regulate membrane protein localization by modulating the ubiquitination process, and they can also cooperate with the ESCRT machinery for protein sorting.^101^ The trans‐oligomerization of Golgi proteins GRASP55 and GRASP65 is essential for maintaining Golgi stacking. Ubiquitination of GRASP55 has been found to target it for proteasomal degradation, which rescues phenotypes such as disrupted Golgi structure, reduced protein secretion, and dendritic branching defects.^102^ Another compelling example is the post‐ER transport of Toll‐like receptors (TLRs). The subcellular localization and intracellular trafficking of TLRs are crucial for the regulation of TLR‐mediated antimicrobial immunity and autoimmune responses. The E3 Ub ligase ring finger protein (RNF)115 inhibits post‐ER transport of TLRs and TLR‐mediated immune responses by catalyzing the ubiquitination of small GTPases RAB1A and RAB13. The 14‐3‐3 chaperone binds to AKT1‐phosphorylated RNF115, promoting its localization to the ER and Golgi apparatus. This indicates that ubiquitination can alter the trafficking of TLRs post‐ER, thereby influencing cellular activities.^103^ Ubiquitination also plays a role when mature proteins leave the Golgi apparatus. Coronin 7 is essential for the budding of transport vesicles derived from the Golgi apparatus, and the Ub ligase Ubr4 impairs the export of secretory proteins from the Golgi by promoting Coronin 7 expression. This function is critical for circadian synchronization and signal processing at the circuit level.^104^ In summary, ubiquitination serves to control protein quality and quantity by participating in ERAD degradation, vesicular transport, and the sorting and processing of proteins within the Golgi apparatus (Figure 3C).

Beyond its role in the trafficking of secretory proteins, ubiquitination also regulates the transport of nuclear proteins. Nuclear localization signals (NLSs) are specific topological amino acid sequences within the protein that can be recognized by import proteins, facilitating their transport into the nucleus. This importin‐mediated nuclear transport mechanism is a promising avenue for therapeutic strategies targeting the nucleus.^105^ In the Nipah virus matrix protein (NiV‐M), a bipartite NLS has been identified that can undergo mono‐ubiquitination to regulate protein export. Ub overexpression enhances NiV‐M budding.^106^

The transcription factor p53 is one of the most commonly mutated tumor suppressors. The mutant form, Δp53, can sequester wild‐type p53, resulting in its retention in the cytoplasm. Δp53 can be ubiquitinated and degraded by mouse double minute 2 homolog (MDM2), indirectly regulating the nuclear localization of p53, thereby affecting tumorigenesis.^107^ Research has found that proteasome inhibition, which leads to the accumulation of ubiquitinated TDP‐43 at lysine 95 within its NLS, reduces poly‐GA‐dependent mislocalization of TDP‐43, offering significant therapeutic potential in amyotrophic lateral sclerosis (ALS) and frontotemporal dementia^108^ (Figure 3D).

Ubiquitinated proteins can directly interact with nuclear transport proteins that possess specific UBDs, such as importins. This interaction facilitates the translocation of ubiquitinated proteins from the cytoplasm to the nucleus. For instance, in renal clear cell carcinoma cells, the subcellular relocalization of circPPAP2B is dependent on the nondegradative ubiquitination of heterogeneous nuclear ribonucleoprotein C (HNRNPC) and the stabilization of the HNRNPC/vimentin/importin α7 ternary complex, thereby promoting cancer cell metastasis. Key Ub enzymes involved in this process include TRIM and USP10.^109^ Ubiquitination also regulates the abundance of plasma membrane receptors and transport proteins, affecting the endosomal degradation of cargoes and the auxin efflux transporter PIN2‐GFP in vivo. In *Arabidopsis*, OTU 11 and OUT12 are plasma membrane‐localized DUBs. They bind phospholipids through multiple motifs in their OTU domains. The DUB activity of OUT11 and OUT12 on K11‐, K6‐, and K63‐linked Ub is stimulated by association with anionic lipid‐containing membranes.^110^

### 3.3 Ubiquitination and signaling pathways

**FIGURE 4:**
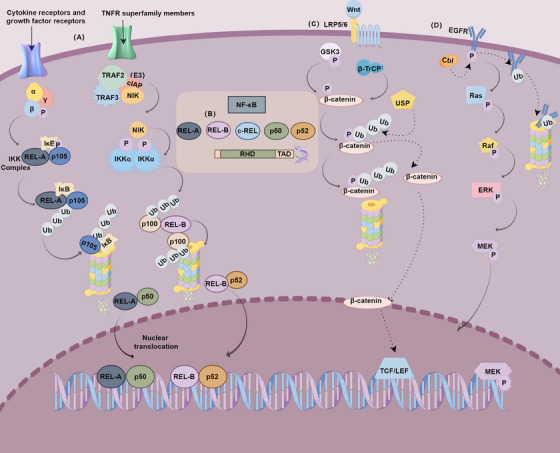
Ubiquitination in key signaling pathways (by Figdraw). (A) Ubiquitination in canonical and noncanonical NF‐κB signaling pathways, regulating the activation and translocation of NF‐κB subunits. (B) Types and structures of NF‐κB, including its various subunits and their functional domains. (C) Ubiquitination in the Wnt‐β‐catenin signaling pathway, influencing the stability and activity of β‐catenin. (D) Ubiquitination in the EGFR signaling pathway, affecting receptor trafficking, degradation, and downstream signaling. TCF, T‐cell factor; LEF, lymphoid enhancer factor; LRP, lipoprotein receptor‐related protein; GSK3, glycogen synthase kinase 3; β‐TrCP, beta‐transducin repeat‐containing protein; RHD, Rel homology domain; TAD, transactivation domains; NIK, NF‐κB‐inducing kinase; cIAP, cellular inhibitor of apoptosis; IKK, IκB kinase; EGFR, epidermal growth factor receptor; P, phosphorylation; Ub, ubiquitin.

The nuclear factor κB (NF‐κB) family comprises transcription factors that play a critical role in various cell responses and are regulated by numerous mechanisms to maintain tolerance and cell homeostasis.^111^ The activation of NF‐κB is intrinsically linked to ubiquitination. Although NF‐κB is widely expressed, it is typically held inactive in the cytoplasm by members of the IκB inhibitory protein family. Rapid degradation of IκB via the Ub–proteasome pathway permits NF‐κB to translocate into the nucleus. The NF‐κB family consists of five members: p65 (REL‐A), c‐REL, REL‐B, p50, and p52, all of which contain the REL homology domain (RHD) essential for DNA binding, dimerization, nuclear localization, and IκB interaction. Additionally, p65, c‐REL, and REL‐B possess transactivation domains (TAD) necessary for gene activation. The precursor proteins p105 and p100, which contain 5 to 7 ankyrin repeats, undergo proteolytic processing upon activation, leading to the generation of p50 and p52, respectively.^111^ When the RHD is masked, NF‐κB cannot translocate to the cytoplasm to exert its functions, with ubiquitination serving as the key to unlocking this inhibition^112^ (Figure 4A,B).

The activation of NF‐κB can be divided into canonical and noncanonical pathways. The canonical pathway mediates the activation of NF‐κB1 p50, REL‐A, and c‐Re, while the noncanonical pathway selectively activates NF‐κB members sequestered by p100, primarily NF‐κB2 p52 and REL‐B. Both activation pathways require ubiquitination. In the canonical pathway, IκB kinase (IKK), composed of α and β subunits and a necessary regulatory subunit γ, phosphorylates IκB. The phosphorylated IκB is then recruited by the F‐box protein beta‐transducin repeat‐containing protein (β‐TrcP) to the Ub ligase complex for polyubiquitination and selective degradation, a process independent of the proteasome. The noncanonical pathway selectively responds to a subset of tumor necrosis factor receptor superfamily members. The pathway involves E3 ligases, particularly cellular inhibitor of apoptosis proteins 1 and 2 (cIAP1/2) targeting NF‐κB‐inducing kinase (NIK) for ubiquitination and regulation. NIK phosphorylates and activates IKKα, which subsequently phosphorylates the carboxy‐terminal serine residues of p100, triggering the selective degradation of the C‐terminal IκB‐like structure of p100, leading to the generation of p52 and the nuclear translocation of p52 and REL‐B.^113^, ^114^ The regulation of NF‐κB activity is also highly dependent on ubiquitination and deubiquitination. For instance, the E3 Ub ligase, PDZ and LIM domain protein 2, inhibits NF‐κB transcriptional activity by removing RelA from DNA‐binding sites and mediating its degradation.^115^ Similarly, A20, encoded by Tnfaip3, is a direct NF‐κB target gene that plays a key role in the negative feedback regulation of canonical NF‐κB. A20 contains a DUB domain and a C2–C2 zinc finger E3 Ub ligase domain. The DUB domain of A20 removes K63‐linked Ub chains from RIP1, TRAF6, and NEMO, leading to the disassembly of the IKK complex and downregulation of the inflammatory response.^116^

The Wnt/β‐catenin signaling pathway plays a pivotal role in embryogenesis and development and is frequently observed in tumorigenesis, particularly in colorectal cancers. The canonical Wnt signaling pathway is activated when Wnt ligands bind to the Frizzled (Fz) family of seven‐transmembrane receptors and their coreceptors, lipoprotein receptor‐related protein (LRP)5/6, on the cell surface, leading to the recruitment of Dishevelled scaffold proteins to the receptor complex. The regulation of cytosolic β‐catenin through protein degradation pathways is the key to Wnt signaling.^117^ The primary E3 Ub ligase responsible for regulating β‐catenin stability is β‐TrCP. Phosphorylation of β‐catenin by glycogen synthase kinase 3 (GSK3) at serines 33 and 37 creates a recognition site for β‐TrCP, which then ubiquitinates β‐catenin, leading to its degradation. Upon translocation into the nucleus, β‐catenin activates T‐cell factor (TCF) and lymphoid enhancer factor (LEF), leading to the transcription of key target genes such as cyclin D1 and c‐Myc.^118^ Loss of RNF43 and ZNRF3 induces rapid growth of adenomas, as RNF43 and ZNRF3 target Wnt receptors for degradation by selectively ubiquitinating Fz receptors, thereby attenuating Wnt signaling.^119^ Conversely, the DUB USP46 complex acts as a positive regulator of Wnt signaling. Wnt signaling promotes the binding of the USP46 complex to the Wnt coreceptor LRP6 at the cell surface, stabilizing LRP6 on the cell surface. This interaction facilitates LRP6 signalosome assembly by removing obstructive Ub chains, which is essential for Wnt‐dependent intestinal organoid viability^120^ (Figure 4C).

Additionally, the Ub ligase Mule inhibits the Wnt pathway by suppressing c‐Myc, thereby controlling unwanted proliferation and stem cell expansion in colorectal cancer.^121^ In the regulation of the intestinal ecosystem, the CUL4B–RING Ub ligase (CRL4B) targets immune‐related GTPase family M member 1 (IRGM1) for proteasomal degradation. The absence of Cul4b results in reduced self‐renewal of intestinal stem cells and diminished lineage differentiation towards secretory progenitor cells through downregulation of Wnt signaling.^122^ Another example of ubiquitination in regulating the Wnt signaling pathway is JADE‐1, which ubiquitinates both phosphorylated and nonphosphorylated β‐catenin. This modulation of β‐catenin stability during both Wnt‐off and Wnt‐on phases supports the involvement of Jade‐1 and Wnt signaling in renal tumorigenesis.^123^ Furthermore, the EGF signaling pathway is deeply involved in ubiquitination processes to maintain protein homeostasis. and itself is also influenced by ubiquitination. In mammalian cells, treatment with EGF leads to rapid modification of ligand‐activated EGFR with K63‐linked Ub chains.^124^ LRIG1, a negative regulator of EGF in mammals, is upregulated and upon EGF stimulation, which is accompanied by enhanced EGFR ubiquitination and degradation. c‐Cbl, an E3 Ub ligase, ubiquitinates both EGFR and LRIG1, leading to their degradation^125^ (Figure 4D).

## 4 UBIQUITINATION IN HEALTH

Ubiquitination, due to its widespread distribution, is implicated in a multitude of biological processes. Its prominent role in regulating protein degradation underscores its importance in maintaining protein quality and quantity, thereby supporting protein homeostasis. The intricate architecture of proteins contributes to their diversity and underscores their significance as fundamental components of living systems. Ub, a small molecule ubiquitously present across various cell types, tags and directs specific proteins for degradation in response to cellular signals. This process facilitates the modification of essential proteins implicated in nuclear DNA repair, cell cycle regulation, and inflammation control. Consequently, ubiquitination emerges as a pivotal regulatory mechanism, contributing to the maintenance of protein homeostasis, DNA repair, cell cycle regulation, and the modulation of inflammatory signaling pathways. This, in turn, establishes a stable foundation for the proper functioning of cellular processes.

### 4.1 Maintenance of protein homeostasis

Protein homeostasis, or proteostasis, is maintained through a complex network of mechanisms that ensure protein quality and support the evolutionary diversity of protein biological functions.^126^ Ubiquitination plays a key role in regulating protein quality, quantity, and spatial localization, achieving a state of equilibrium for these proteins.^127^ Protein imbalance can lead to abnormal aggregation of normal or aberrant proteins, triggering a range of related diseases and aging.^128^ Proteins, with their diverse structures and functions, are distributed throughout the cell and perform different roles by altering their abundance, distribution, and activity. Proteostasis can be categorized into maintenance under normal and stress conditions.^129^, ^130^ To adapt to various cellular states and external environments, protein homeostasis under normal conditions is reflected by the maturation of proteins through other types of PTMs. Under stress conditions, the UPS mediates the proteolysis of soluble ubiquitinated proteins (in the presence of chaperones) that are misfolded, oxidized, mutated, or otherwise damaged, predominantly participating in the organism's self‐protection mechanism.^131^ For instance, Ub inhibits the maturation of amyloid precursor protein (APP) by sequestering it in early secretory pathways, primarily within the Golgi apparatus. This sequestration significantly delays the proteolytic processing of APP by secretases and the proteasome, which is crucial for the onset of late‐onset Alzheimer's disease (AD).^132^ Additionally, under acute ER stress, prion proteins are prevented from mislocalizing to the ER and are directed for cytosolic degradation, minimizing prion protein secretion and benefiting the cell. This degradation is attributed to the UPS.^133^ The deeper mechanism involves the disposition of such mislocalized proteins depending on the BAG6 complex (such as BAG6, TRC35, and UBL4A). The BAG6 complex recognizes mislocalized proteins, recruits the E2 conjugating enzyme UbcH5 and an unidentified E3 ligase, thus selectively promoting their rapid ubiquitination and proteasomal degradation.^134^ Beyond degrading harmful proteins under stress, ubiquitination also degrades excess normal proteins, contributing to cellular homeostasis. Many proteins function within multisubunit complexes requiring proper assembly, and the degradation of unassembled soluble proteins (termed unassembled soluble protein degradation, USPD) necessitates the Ub‐selective chaperone p97, its cofactor nuclear protein localization protein 4 (Npl4), and the proteasome. HUWE1, a protein containing domains homologous to the E6‐AP carboxyl terminus, serves as the Ub ligase for substrates with exposed hydrophobic regions.^135^ As previously mentioned, ERAD is another way to control protein homeostasis, capable of degrading functional proteins such as rate‐limiting metabolic enzymes. An example of ERAD controlling ligand‐dependent abundance is the feedback regulation of 3‐hydroxy‐3‐methylglutaryl‐CoA reductase, a key enzyme in the cholesterol biosynthesis pathway.^136^ Among the myriad mechanisms maintaining protein homeostasis, modulation through ubiquitination is a relatively late stage of regulation, directly controlling protein levels. Researchers have found that the regulation of epidermal growth factor (EGF) signaling is associated with mechanisms that maintain protein homeostasis. EGF signaling shifts the strategy of maintaining protein homeostasis from a chaperone‐based approach to one involving enhanced UPS activity and polyubiquitination, thereby reducing protein aggregation.^137^ Therefore, although multiple strategies are required to maintain protein homeostasis, regulation by the UPS is a more direct method of controlling protein quantity.

### 4.2 Role in DNA repair and genome stability

DNA double‐strand breaks (DSBs) are one of the most severe types of DNA damage. In response, mammals have evolved complex cellular pathways to facilitate DNA repair and maintain genome stability: the homologous recombination (HR) and nonhomologous DNA end joining pathways.^138^ During DSBs, the breast cancer type 1 (BRCA1)–BRCA1‐associated RING domain protein 1 (BARD1) complex, which possesses E3 ligase activity, aids in DSB repair through HR by promoting nucleolytic resection at DNA ends.^139^ The BRCA1–BARD1 complex localizes to damaged chromatin post‐DNA replication and catalyzes the ubiquitination of histone H2A and other cellular targets. The RING domains within BRCA1–BARD1 orient the E2 Ub‐conjugating enzyme atop nucleosomes in a dynamic conformation, preparing for the transfer of Ub to the flexible C‐terminal tails of H2A and its variant H2AX. Concurrently, the nuclear E3 Ub ligase RNF168 rapidly ubiquitinates histone deacetylase 6 (HDAC6) at lysine 116, targeting it for degradation. Under the modification by RNF168, ubiquitinated lysine 15 on H2A binds to the DNA repair protein 53BP1, thus inhibiting 53BP1 and promoting HR repair of DSBs.^140^, ^141^, ^142^, ^143^ Moreover, the nucleolytic resection induced by BRCA1–BARD1 also recruits another tumor suppressor complex, BRCA2–PALB2, and the recombinase RAD51 to single‐strand DNA templates, further facilitating DSB repair.^139^ HR repair of DSBs is also mediated by MDC1. RNF8 and RNF168 assist DNA repair proteins in reaching sites of DNA damage, and these two E3 ligases are connected by Lethal (3) malignant brain tumor‐like protein 2 (L3MBTL2). MDC1 recruits this crucial factor, which is subsequently ubiquitinated by RNF8, and the ubiquitinated L3MBTL2 then recruits RNF168.^144^ Ubiquitination‐associated DNA repair through recombination is also linked to the cell cycle. CDK2 can phosphorylate RNF4 at T26 and T112, enhancing RNF4 E3 ligase activity, which is important for MDC1 degradation during the S phase and proper HR repair.^145^

Fanconi anemia (FA) is a prototypical disease associated with DNA crosslink damage. The excision of “toxic replication” intermediates is the key to the pathogenesis of FA, which is also pivotal for the repair of DNA interstrand crosslinks.^146^ Within the FA network, FANCD2 and FANCI function together to facilitate the repair of DNA crosslinks. These proteins form a DNA‐binding heterodimeric ID2 complex and undergo mono‐ubiquitination when cells are exposed to DNA crosslinking agents.^147^ Studies have shown that Ub is positioned at the interface of FANCD2 and FANCI, acting as a molecular pin to capture the closed conformation of ubD2‐I (a critical factor in DNA crosslink repair) clamped onto the DNA. Thus, the mono‐ubiquitination of the FANCD2–FANCI heterodimer is a critical step in the FA DNA crosslink repair pathway.^148^ The interaction between FANCD2 and FANCI within the heterodimer protects monoubiquitinated FANCD2 from polyubiquitination and subsequent proteasomal degradation.^149^ RAD18, an E3 ligase, binds to FANCD2 and is essential for the effective mono‐ubiquitination and chromatin localization of both FANCD2 and FANCI. RAD18 facilitates the recruitment of FANCI and FANCD2 to chromatin and their mono‐ubiquitination during the S phase.^150^

**FIGURE 5:**
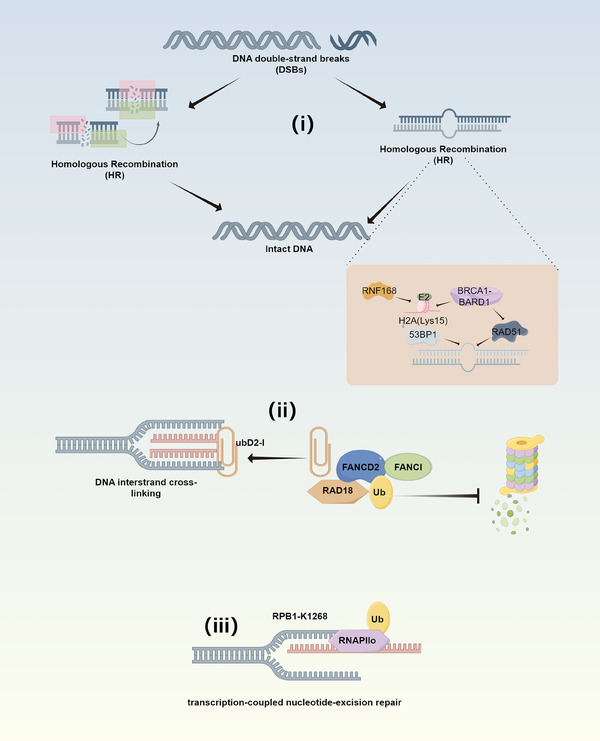
Ubiquitination in DNA repair (by Figdraw). (i) Involvement in DNA double‐strand break repair; (ii) role in DNA crosslink repair; (iii) participation in transcription‐coupled nucleotide excision repair. RNF, ring finger protein; BRCA1, breast cancer type 1; BARD1, BRCA1‐associated RING domain protein 1; Ub, ubiquitin.

In addition to its role in repairing DNA DSBs, ubiquitination is also involved in the repair of other types of DNA damage, such as transcription‐coupled nucleotide excision repair (TC‐NER). TC‐NER is initiated when elongating RNA polymerase II (RNAPIIo) stalls at sites of DNA damage. In the UV‐sensitive syndrome (UVSS), the UVSSA protein interacts with the TC‐NER mechanism and stabilizes the ERCC6 complex (associated with Cockayne syndrome genes). UVSSA also promotes the ubiquitination of RNAPIIo stalled at DNA damage sites, thereby initiating the TC‐NER process.^151^ A single DNA damage‐induced ubiquitination site has been identified on RNAPIIo at RPB1‐K1268, which plays a critical role in regulating transcription recovery and DNA damage resistance. The ubiquitination of RPB1‐K1268 is crucial for the repair of DNA damage and resolving transcriptional stalling. Mice with a knock‐in mutation at RPB1‐K1268R exhibit shortened lifespans, premature aging, and neurodegeneration.^152^ NER is another significant pathway for DNA damage repair that influenced by ubiquitination. DNA–protein crosslinks (DPCs) are large cytotoxic DNA lesions formed following exposure to chemotherapeutic agents and environmental chemicals. In cells proficient in NER, DPCs undergo K48‐linked polyubiquitination and are removed via a proteasome‐dependent mechanism, whereas in NER‐deficient cells, DNA‐conjugated proteins undergo K63‐linked ubiquitination.^153^ Furthermore, recent studies have indicated that members of the SWI/SNF and INO80 families, along with poly (ADP‐ribose) polymerase 1 (PARP1), are involved in NER. The H2A‐Ub‐binding protein ZRF1 and the endonuclease DICER influence chromatin conformation through PARP1. Overall, Ub signaling cascades are closely associated with chromatin functions.^154^ Besides Ub cascades, deubiquitination also plays a role in regulating genomic stability. For example, the deubiquitinase USP15 interacts with and deubiquitinates PARP1, promoting its stability and thereby stimulating DNA repair, genomic stability, and the proliferation of triple‐negative breast cancer cells.^155^ In the context of mismatch repair (MMR), the proteasomal degradation of cyclin‐dependent kinase (CDK) inhibitor p21, which competes with MMR proteins for binding to proliferating cell nuclear antigen, facilitate MMR. This mechanism primarily operates during the G1/S transition, where the timely cullin–RING Ub ligase (CRL)‐dependent degradation of cyclin D and p21 allows for effective MMR activity to correct DNA replication errors^156^ (Figure 5).

### 4.3 Regulation of cell cycle progression and proliferation

The primary drivers of cell cycle progression are the sequential activations of CDKs, which are regulated, in part, through the Ub‐mediated proteolysis of their cyclin partners and kinase inhibitors (CKIs). Studies have shown that in eukaryotic cells, the TCR/cyclosome (APC/C) is responsible for the ubiquitination and subsequent proteasomal degradation of many CDK regulatory factors, ensuring that the cell cycle proceeds in a timely and precisely regulated manner.^157^

In eukaryotes, the APC/C is a multisubunit E3 Ub ligase that regulates the Ub‐independent proteolysis of specific cyclins at different stages of mitosis to coordinate chromosome segregation and transition into G1. During mitosis, CDKs and polo kinase control the activation of APC/C mediated by cell‐division cycle protein 20 homologue (Cdc20) and E‐cadherin gene (Cdh1).^158^, ^159^ Inhibition of Cdc20 phosphorylation leads to premature activation of APC/C‐Cdc20, causing instability in several substrates, including cyclin B1 and A2, resulting in an extended G2 phase and delayed entry into mitosis.^160^ Studies have identified K11‐linked Ub chains as important signaling entities in cell cycle control. Their efficient formation depends on specific interaction between E2 Ub enzyme UbcH10 and APC/C. The APC/C targets cell cycle proteins for degradation by the 26S proteasome. During the G1 phase, APC/C inactivation involves the degradation of its specific E2 UbcH10, which assembles K11‐linked chains.^55^ Upon entry into the M phase, the silencing of the spindle assembly checkpoint (SAC) activates the Ub ligase APC/C, leading to the proteasomal destruction of the separase inhibitor securin and cyclin B. This destruction releases separase, which cleaves cohesion, thereby facilitating chromosome segregation.^161^ The SAC prevents the APC/C from recognizing cyclin B and securin by incorporating of the APC/C coactivator CDC20 into a complex known as the mitotic checkpoint complex. The SAC generates a diffusible “wait anaphase” signal through unattached kinetochores.^162^ In securin‐deficient cells, researchers have identified human shugoshin 2 (Sgo2), which forms a complex with mitotic‐arrest deficient‐1 (Mad2), substituting the role of securin in these cells.^163^ The interplay between APC/C, the SAC, and early mitotic inhibitor 1 (Emi1) creates a system of mutual checks that determines whether a cell proceeds to the next cell cycle.^164^ Additionally, the CDK inhibitor p27Kip1 is exported from the nucleus to the cytoplasm during G0–G1 transition and is degraded via the Ub–proteasome pathway at this stage.^165^

In addition to the large E3 Ub complex APC/C, other Ub‐related molecules also play crucial roles in regulating the cell cycle. Efficient transitions in the cell cycle are crucial for embryonic development and tissue homeostasis, particularly at the G1/S and G2/M checkpoints. The G1/S checkpoint ensures that the cell's condition and nutritional status are optimal before entering the S phase. The PARK2 E3 Ub ligase, a tumor suppressor, coordinately control the stability of cyclin D and cyclin E, acting as a primary regulator of G1/S cyclin stability.^166^ In MCF‐7 cells, HDAC inhibitors such as trichostatin A enhance cyclin D1 degradation through a GSK3β/CRM1‐dependent nuclear export and 26S proteasome degradation pathway.^167^ Ubiquitination also indirectly regulates the cell cycle by modulating cell cycle inhibitors like p21. Endogenous DNA damage occurring in the S phase leads to p53‐dependent accumulation of p21 during the subsequent G2 phase of the mother cell and G1 phase of the daughter cell, regulating the proliferation‐quiescence decision of daughter cells through CDK2 inhibition. Sub‐threshold accumulation of p21 does not affect the G1/S transition. The Ub ligases CRL4^Cdt2^ and SCF^Skp2^ couple the degradation of sub‐threshold p21 prior to the G1/S transition, thereby ensuring an irreversible G1/S transition.^168^ Moreover, the transcription factor nuclear casein and cyclin‐dependent kinase substrate 1 (NUCKS1) is recruited to chromatin to activate the expression of S‐phase kinase‐associated protein 2 (SKP2), the F‐box component of the SCFSKP2 Ub ligase, leading to the degradation of p21 and p27 and promoting progression to the S phase.^169^

There is also a checkpoint between the G2 and M phases to ensure that all necessary conditions are met before the cell enters mitosis, with ubiquitination playing a crucial role in controlling the progression of mitosis. During centriole replication, USP33 deubiquitinates CP110, leading to CP110 instability, thereby inhibiting centrosome amplification and preventing mitotic defects. This mechanism primarily operates during the S and G2/M phases, the periods of centriole replication and elongation.^170^ Furthermore, the human single‐stranded DNA binding protein SSB1 (hSSB1) is a novel DNA damage‐associated protein that can interact with p53 and protect p53 from Ub‐mediated degradation. Consequently, the inactivation of hSSB1 leads to G2/M checkpoint failure.^149^

The Golgi apparatus disassembles and disperses its stacks at the onset of mitosis, followed by further vesiculation. The Ub ligase activity of HACE1 during Golgi disassembly in mitosis is essential for subsequent Golgi membrane fusion postmitosis. Depleting HACE1 using small interfering RNA or the expressing inactive HACE1 mutant proteins in cells impairs Golgi membrane fusion after mitosis.^171^ The disassembly of integrin‐containing focal adhesions is crucial for cell rounding, the formation of mitotic retraction fibers, bipolar spindle positioning, and chromosome segregation. The underlying mechanism involves the phosphorylation of the integrin activator kindlin by the CDK1–cyclin B1 complex, which subsequently leads to the recruitment of the Cullin 9–FBXL10 Ub ligase complex that mediates kindlin ubiquitination and degradation.^172^

**FIGURE 6:**
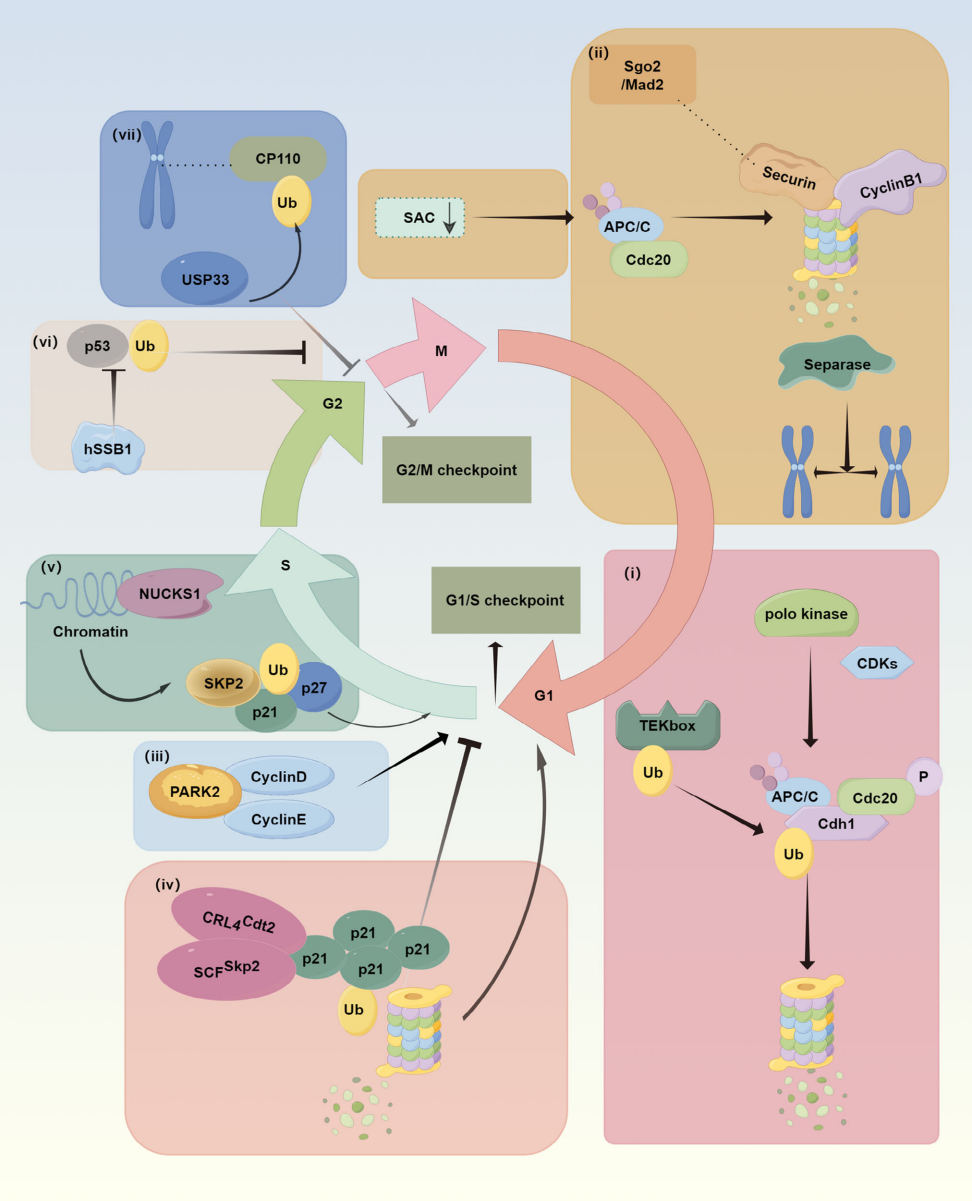
Ubiquitination in cell cycle regulation and genome stability (by Figdraw). (i and ii) APC/C in cell cycle regulation; (iii–v) ubiquitination in the regulation of G1/S transition; (vi and vii) ubiquitination in the regulation of G2/M transition. SAC, spindle assembly checkpoint; Sgo2, shugoshin 2; Mad2, mitotic‐arrest deficient‐1; hSSB1, human single‐stranded DNA binding protein SSB1; NUCKS1, nuclear casein and cyclin‐dependent kinase substrate 1; SKP2, S‐phase kinase‐associated protein 2; APC/C, anaphase‐promoting complex/cyclosome; CDKs, cyclin‐dependent kinases; Cdc20, cell‐division cycle protein 20 homologue; Cdh1, E‐cadherin gene; P, phosphorylation; Ub, ubiquitin.

In summary, the checkpoints at various stages of the cell cycle are critical for cell cycle regulation. Based on this, Sakaue‐Sawano et al.^173^ developed Fucci (fluorescent ubiquitination‐based cell cycle indicator), a genetically encoded optical sensor for monitoring interphase in the cell cycle. Its principle relies on S‐phase‐specific CUL4Ddb1‐mediated ubiquitination to precisely distinguish major cell cycle transitions and phases, particularly G1, S, and G2 (Figure 6).

### 4.4 Importance in immune response and inflammation

During the initiation of immune responses, ubiquitination mediates various immune processes through interactions with multiple receptors. For instance, Nrdp1, a RING‐type E3 ligase, mediates K33‐linked polyubiquitination of the signaling kinase Zap70 while promoting dephosphorylation of Zap70 by Sts1 and Sts2, thereby blocking TCR signaling in CD8+ T cells.^174^ The Ub E3 ligases Itch and WW domain‐containing protein 2 (WWP2) functionally collaborate in regulating CD4+ TCR signal strength. They form a complex and synergistically enhance proximal TCR signal strength by catalyzing the conjugation of atypical Ub chains to the phosphatase SH2 domain‐containing protein tyrosine phosphatase 1 (Shp‐1), reducing SHP‐1′s association with the tyrosine kinase Lck.^175^ SHARPIN, as part of the LUBAC, does not directly regulate TCR signal strength; However, SHARPIN deficiency leads to a reduction in the number and function of regulatory T cells, independent of NF‐κB signaling.^176^ UBR2, an E3 Ub ligase, acts as a positive regulator of T cell activation. It induces K63‐linked ubiquitination at Lys99 and Lys276 of kinase Lck, which subsequently activates Lck through Tyr394 phosphorylation, thereby promoting T cell activation.^177^

T cell homeostasis is a crucial factor in maintaining immune balance. A typical example of its dysregulation involves the deubiquitinase USP8, which has been identified as a new component of the TCR signalosome, interacting with the adaptor molecule GADS and 14‐3‐3 β. Mice with a knockout of the USP8 gene exhibit lethal colitis.^178^ In the pathogenesis of allergic asthma, TCR signaling can upregulate the levels of the deubiquitinase USP38, which, in turn, stabilizes the TH2 development factor JunB, ultimately promoting asthma.^179^ Ubiquitination processes also influence T cell differentiation. Ndfip1, an activator of the Nedd4 family E3 ligases, regulates cytokine signaling by mediating the degradation of Jak1, thereby limiting the expansion and function of CD4+ effector T cells.^180^

In the activation and differentiation of B cells, the ubiquitination process plays a crucial role in regulating the function of E3 ligases Cbl and Cbl‐b (collectively known as Cbls). Ablation of these ligases impairs the clonal expansion of high‐affinity germinal center (GC) B cells, primarily due to an early exit from the GC cycle. Cbls are highly expressed in the light zone of the GC and impede plasma cell differentiation by promoting Irf4 ubiquitination.^181^ Smurf2 mediates the ubiquitination and degradation of Yin Yang 1 (YY1), a critical GC transcription factor. Smurf2 deficiency enhances YY1‐mediated transactivation of c‐Myc and B cell proliferation, which can also lead to lymphoma in proliferating B cells.^182^ The E3 Ub ligase Itch functions within B cells to limit the numbers of naive and, to a greater extent, GC B cells.^183^ The surface levels and degradation of MHCII on GC B cells are dynamically regulated, with fluctuations in surface MHCII levels dependent on ubiquitination and the E3 ligase March1.^184^

In inflammatory responses, activator protein‐1 (AP‐1) is a key regulator that can both promote inflammation and cell damage, as well as enhance cellular antioxidant capacity to protect cells from damage. Ubiquitination of AP‐1 is a classical pathway in the regulation of inflammatory mechanisms.^185^ As an E3 Ub ligase, TRIM5 enhances innate immune signaling through interaction with retroviral capsid lattices, promoting UBC13–UEV1A‐dependent E3 activity, activating the TAK1 kinase complex, and stimulating AP‐1 and NF‐κB signaling. Additionally, TRIM5 also acts as a pattern recognition receptor, specifically recognizing retroviral capsid lattices and promoting the transcription of AP‐1 and NF‐κB‐dependent factors.^186^ Another member of the TRIM family, TRIM7, mediates c‐Jun/AP‐1 activation through Ras signaling. The specific mechanism involves the stabilization of the AP‐1 coactivator RACO‐1 via K63‐linked ubiquitination, thereby promoting AP‐1‐dependent gene expression.^187^ Furthermore, DR5 inhibits signaling by promoting sphingosine‐1‐phosphate‐dependent polyubiquitination of TRAF2, which activates the JNK/AP‐1 signaling pathway.^188^ These mechanisms illustrate the multifaceted roles of ubiquitination in regulating AP‐1 activity, involving not only direct signal transduction activation but also the stabilization of coactivators and the regulation of downstream signaling pathways, collectively forming a complex regulatory network of ubiquitination in inflammatory responses. Additionally, canonical NF‐κB is rapidly activated in innate and adaptive immune cells through various signals, and its regulation of inflammatory responses has been detailed in previous pages.

**FIGURE 7:**
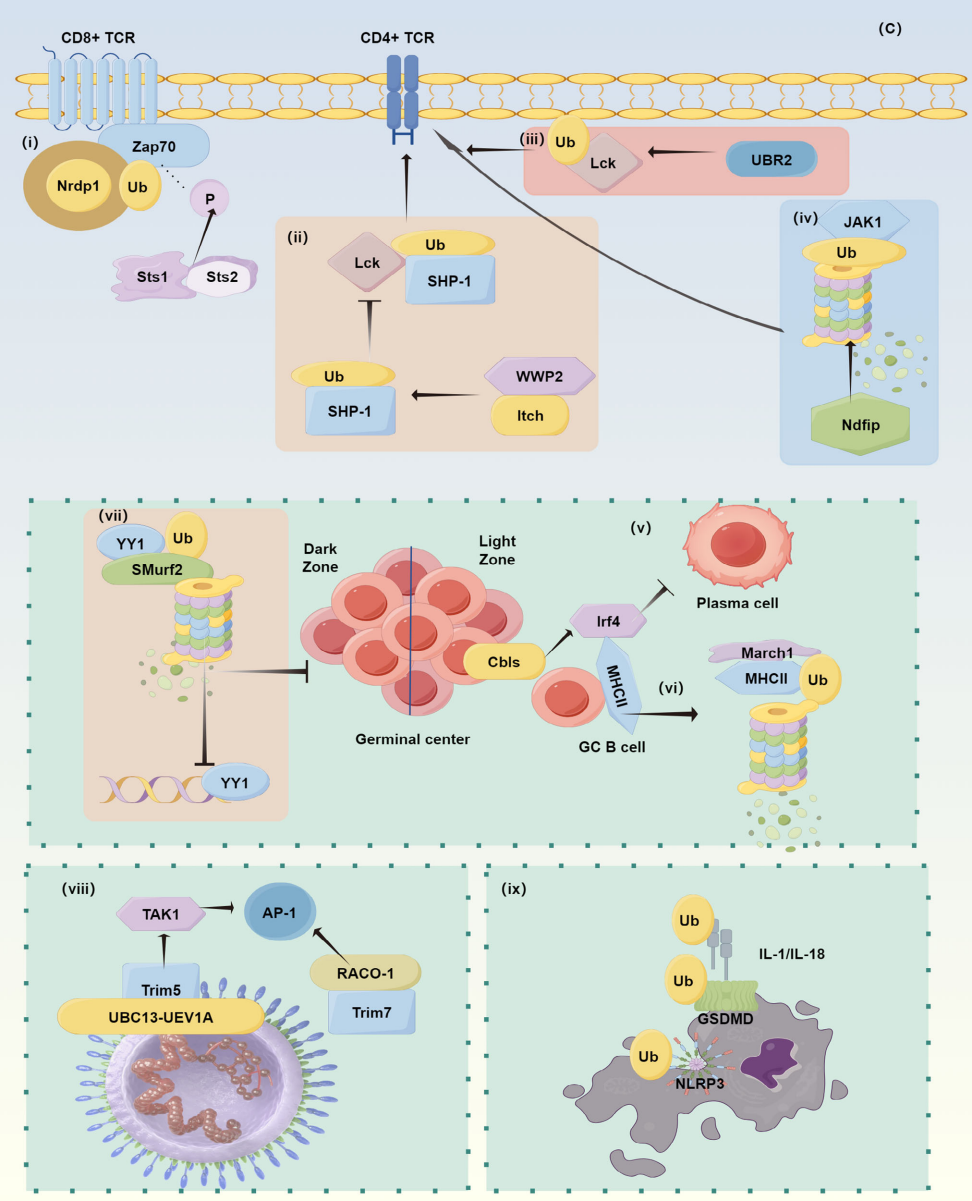
Ubiquitination in inflammation regulation (by Figdraw). (i–iv) Ubiquitination and TCR signaling; (v–vii) ubiquitination in B cell differentiation; (viii) ubiquitination in conjunction with AP‐1 in inflammation regulation; (ix) ubiquitination in cell death (pyroptosis). TCR, T cell receptor; Shp‐1, SH2 domain‐containing protein tyrosine phosphatase 1; WWP2, WW domain‐containing protein 2; YY1, Yin Yang 1; AP‐1, activator protein‐1; GSDMD, gasdermin D; P, phosphorylation; Ub, ubiquitin.

Programmed cell death can influence the release of inflammatory factors. There are multiple forms of programmed death, including apoptosis, pyroptosis, and necroptosis, each accompanied by the release of inflammatory factors.^189^ For instance, pyroptosis is a form of programmed cell death triggered by inflammasome activation, leading to the formation of pores in cell membranes by gasdermin D (GSDMD), ultimately resulting in the release of cellular contents and the release of inflammatory factors.^190^ Ubiquitination plays a critical role in modifying multiple molecules involved in cell death. In the canonical pyroptosis pathway, ubiquitination is involved at every stage, from the initial extracellular stimuli acting on the NLRP family to the ultimate release of inflammatory factors. The NLRP3 inflammasome, the most common initiator of pyroptosis, consists of NLRP3, ASC, and activated caspase‐1.^191^ Ubiquitination of the NLRP3 inflammasome generally inhibits its assembly and activation, while deubiquitination often has the opposite effect.^192^, ^193^ GSDMD, the executor of pyroptosis, when ubiquitinated, often promotes the occurrence of pyroptosis.^194^ Even inflammatory factors themselves, such as the IL‐1β, can be ubiquitinated, undergoing various types of ubiquitination.^195^ Overall, ubiquitination indirectly but commonly plays a regulatory role in inflammatory responses by modifying molecules involved in cell death (Figure 7).

## 5 UBIQUITINATION IN DISEASE

Ubiquitination plays a crucial role in a wide range of biological processes, throughout the human body. Proteins are essential components of human physiology, with different proteins performing specific functions, intricately linked in a sequential manner to regulate diverse biological pathways. When the ubiquitination process of a critical protein is dysregulated, it can severely impact the multitude of cellular processes. Such dysregulation can escalate to a point where disease is triggered. Currently, extensive research has focused on diseases arising from the aberrant regulation of ubiquitination, including various cancers, neurodegenerative diseases, and metabolic disorders. The following sections will systematically elaborate the role of ubiquitination dysregulation in various disease contexts.

### 5.1 Dysregulation of ubiquitination in cancer

Aberrant activation or dysregulation of ubiquitination can lead to abnormal pathway activation, improper assembly of protein complexes, and the accumulation of misfolded proteins, all of which can disrupt normal physiological functions and contribute to disease pathogenesis. The complexity and diversity of the ubiquitination process determine its varying roles in different physiological and pathological contexts, playing a critical role in maintaining cellular functions, growth, differentiation, and immune defense.^196^, ^197^, ^198^ Recent studies have indicated that ubiquitination is also extensively involved in the processes of cell death and inflammatory responses. In fact, the scope of the Ub system is still under investigation, as the reactions and signaling processes it influences are likely far more extensive than currently understood. There are approximately 100 specialized proteases, known as DUB, within the human body that can counteract the intricate ubiquitination process. In this context, the balance between E3 ligases and DUBs is a crucial for ensuring the proper functioning of signaling pathways and cellular functions.^199^ This delicate interplay between ubiquitination and deubiquitination is likely pivotal in the progression of diseases, including cancer, and could represent a breakthrough in the development of therapeutic interventions.

Compared to normal cells, cancer cells exhibit accelerated proliferation and require significantly higher metabolic activity to support their malignant growth. Dysregulation of metabolic activities is a significant contributor to tumorigenesis. Among the numerous PTMs closely related to metabolic activities, protein ubiquitination stands out as a common and crucial cellular mechanism. Dysregulation of ubiquitination and deubiquitination has been observed in various types of cancer.^200^, ^201^, ^202^ Moreover, the UPS is regulated by transcriptional, translational, and PTMs, thereby playing various roles in both the promotion and inhibition of oncogenesis. In recent years, owing to the extensive involvement of the UPS in the initiation and progression of cancer, it has garnered widespread attention from researchers as a novel therapeutic target for cancer treatment.^14^

The UPS is extensively involved in the regulation of various cancer‐related signaling pathways, transcription factors, and metabolic enzymes. Within this broad physiological context, the binding processes of E3 ligases and DUBs with their substrates are notably complex. This complexity arises because a single E3 Ub ligase or DUB can target multiple substrates, while an individual substrate protein can be regulated by multiple E3 ligases or DUBs.^202^ For example, FBXW7, an E3 ligase for the crucial oncogene c‐Myc, can function as a tumor suppressor by targeting mTOR, HIF‐1α, c‐Myc, and SREBP1 for degradation.^203^, ^204^, ^205^ Additionally, in response to DNA damage, FBXW7 can mediate the proteasomal degradation of p53, leading to radioresistance.^206^ Moreover, the processes of ubiquitination and deubiquitination in cancer are highly context dependent. For instance, in certain environments, DUBs may exhibit pro‐tumorigenic effects, while in other distinct environments, their tumor‐inhibitory roles become more pronounced. This variability and disparity underscore the significance of targeting specific DUBs for cancer therapy.^199^

The UPS is extensively involved in various biological processes in tumor cells, such as proliferation, invasion, and apoptosis. For instance, homologous to the E6‐associated protein carboxyl terminus domain containing 3, acting as a prosurvival protein, can promote the stabilization of MALT1 via K63‐linked polyubiquitination, ultimately leading to cancer cell proliferation and invasion.^207^, ^208^ Furthermore, FBXW7, identified as a tumor suppressor, interacts with Mcl‐1, prompting the ubiquitination and degradation of this Bcl‐2 family member, thereby inducing apoptosis in cancer cells.^209^ From a cancer‐type perspective, dysregulation of the UPS also influences the growth, proliferation, and metastasis of various cancers. For example, studies have shown that ATXN3 can promote breast cancer metastasis by deubiquitinating KLF4.^210^ Moreover, the lncRNA LNC473 can enhance the proliferation and invasion of liver cancer cells by interacting with USP9X to inhibit the ubiquitination of survivin.^211^ Additionally, in renal cell carcinoma, an E3 ligase encoded by the TRC8 gene interacts with and ubiquitinates heme oxygenase‐1, which in turn enhances the tumorigenic and invasive capabilities of renal cancer cells. In prostate and gastric cancers, RBX1 targets CYCLIN E1, promoting tumor cell proliferation.^212^, ^213^ These examples illustrate the complex and extensive roles of the UPS in cancer. The UPS not only regulates protein degradation but also influences the interaction of proteins with other molecules, thus modulating intracellular signaling networks. In cancer, aberrant expression or mutations of certain E3 ligases can alter cellular responses to specific signaling pathways, leading to uncontrolled cell cycles and enhanced antiapoptotic capabilities. For instance, NEDD4‐1, an E3 Ub ligase, negatively regulates the PI3K/AKT signaling pathway by ubiquitinating and promoting the degradation of PTEN, which facilitates tumor cell proliferation in some cancers, such as lung cancer.^214^ Furthermore, APC/C, an important E3 ligase, can activate Cdc20 to regulate β‐catenin, promoting the activation of the WNT/β‐catenin pathway associated with prostate cancer progression.^215^ These examples further demonstrate the complexity and diversity of ubiquitination pathways in different cancer types, providing new insights and methods for cancer therapy.

Deubiquitination also exerts considerable influence in cancer biology (Table 1). DUBs can regulate the E3 ligase complexes or modulate the signaling cascades that induce protein degradation, thereby impacting the overall balance between ubiquitination and deubiquitination. This action is not limited to a single target but can affect a multitude of substrate proteins. Normally, these processes are precisely controlled to ensure the proper functioning of physiological processes. However, during cancer progression, this delicate balance can be disrupted. The complexity of deubiquitination in cancer stems from its involvement in nearly every aspect of oncogenesis. DUBs target a wide array of proteins involved in numerous cancer‐related signaling pathways, and a single DUB can also exhibit different roles depending on the tumor type and context—roles that may be either oncogenic or tumor‐suppressive.^199^ Like the ubiquitination process, DUBs are also involved in cancer metastasis and proliferation, with different DUBs exerting varying effects on the disease. The research by Han et al. provides a detailed exposition of the roles of different DUBs in promoting and inhibiting cancer.^216^ Nevertheless, targeting the key enzymes in ubiquitination and deubiquitination processes may represent a novel and critical approach to cancer therapy. In summary, dysregulation of the ubiquitination pathway in cancer affects not only the stability of tumor‐associated proteins but can also profoundly influence the disease by altering cell signaling and cell cycle regulation. Future research should continue to elucidate the specific mechanisms of the UPS in different types of cancer and develop targeted therapeutic strategies, providing new directions and methods for cancer treatment.

**TABLE 1:** Inhibitor drugs targeting UPS and DUBs for cancer treatment.

UPS and DUBs	Anticancer drugs/inhibitor	References
Proteasome	Bortezomib, curcumin, b‐AP15	^217^, ^218^, ^219^, ^220^, ^221^, ^222^, ^223^
Ubiquitin proteasome system	Piperlongumine, curcusone D	^220^, ^223^
USP1	ML323, SJB2‐043, SJB3‐019A, GW7647, pimozide, rottlerin	^224^, ^225^, ^226^
USP2	ML364, LCAHA, 6TG	^227^, ^228^
USP4	Vialinin A	^229^, ^230^
USP5	WP1130, vialinin A	^229^, ^230^, ^231^, ^232^
USP7	FT671, FT827, GNE‐6640, GNE‐6776, compound 1, compound 4, P5091, P22077, HBX41108, HBX19818, Cpd1, Cpd2, Cpd8	^231^, ^233^, ^234^, ^235^, ^236^, ^237^, ^238^, ^239^
USP8	DUBs‐IN‐2, MB7295	^240^
USP9X	WP1130, EOAI3402143	^233^, ^241^
USP11	MIX	^242^
USP14	IU‐1/analogues, b‐AP15, VLX1570, WP1130, auranofin	^233^, ^243^, ^244^, ^245^, ^246^
USP15	MIX	^242^
USP17	WP1130	^233^
USP20	GSK2643943A	^247^
USP24	EOAI3402143	^241^
USP25	AZ1/2/3/4	^248^
USP28	AZ1/2/3/4	^248^
USP30	FT385, MF094, MF095	^249^
USP47	Compound 1	^239^
POH1	8‐Thioquinoline, capzimin, phen	^247^, ^250^, ^251^
UCHL1	LDN‐57444, GK13S, 6RK73	^252^, ^253^
UCHL5	IU‐1/analogues, b‐AP15, VLX1570, WP1130, auranofin	^231^, ^243^, ^244^, ^245^, ^246^

### 5.2 Neurodegenerative diseases and protein aggregation

The UPS is responsible for degrading the majority of proteins in the human body and influences biological activities such as cell fate, differentiation, and migration, all of which are crucial for the nervous system. Neurodegenerative diseases, including AD, Parkinson's disease (PD), Huntington's disease (HD), and ALS, are characterized by the accumulation of abnormal proteins and the formation of inclusion bodies.^254^ In these diseases, dysfunction of the UPS is a critical factor leading to the accumulation and aggregation of aberrant proteins, such as β‐amyloid (Aβ) and α‐synuclein. Under normal conditions, the UPS is highly selective, maintaining the stability of the nervous system by degrading short‐lived, abnormal, or misfolded proteins. However, genetic factors, aging, or lifestyle changes can disrupt this process, leading to the accumulation of toxic protein aggregates and the emergence of pathological features associated with neurodegenerative diseases.^255^ When the UPS malfunctions, the misfolding and aggregation of proteins not only impair neuronal function but also trigger intracellular stress responses, ultimately leading to cell death.^256^ Studies have shown that protein aggregates in the brains of many patients with neurodegenerative diseases are Ub positive.^257^ For example, the accumulation of Aβ plaques and hyperphosphorylated tau are prominent pathological features of AD. Research indicates that the proteasome extensively regulates the production of these proteins, and inhibiting proteasome function leads to their abnormal accumulation.^258^ Additionally, the aggregation of huntingtin protein is a significant pathological marker of HD. These aggregates, which should be marked and degraded by ubiquitination, cannot be digested by the proteasome, resulting in their accumulation.^259^ Recent studies in PD have found that the UPS also accelerates the pathological progression of PD by regulating α‐synuclein misfolding and aggregation, mitophagy, neuroinflammation, and oxidative stress.^260^ Furthermore, the UPS influences protein quality control and the maintenance of the intracellular environment, participating in the regulation of mitochondrial function. Mitochondrial homeostasis is closely related to the UPS, as the UPS regulates organelle dynamics, mitochondrial proteome, and mitophagy.^261^ Numerous studies have confirmed that UPS abnormalities may contribute to the progression of PD through mitochondrial dysfunction.^262^, ^263^, ^264^ ALS is a progressive fatal disease, and its rare early‐onset familial form can be caused by mutations in the superoxide dismutase (SOD1) gene. However, researchers have found that the pathological changes are not due to altered SOD1 activity but rather the accumulation of ubiquitinated aggregates, indicating that UPS dysfunction is involved in the progression of ALS. The UPS is a critical component of the protein quality control system, and targeting the UPS may represent a promising approach for treating neurodegenerative diseases.

Although deubiquitination is the opposite of ubiquitination, DUBs are one of the various checkpoints that ensure the correct ubiquitination of substrates. By removing Ub, DUBs are involved in multiple physiological processes of the nervous system, including neuronal fate determination, axon guidance, synaptic communication, and plasticity.^265^ Certain DUBs, such as ataxin‐3, can enhance the degradation of specific proteins by editing the Ub chains attached to substrates. DUBs like USP7 and USP9X can stabilize proteasome substrates by counteracting the activity of certain E3 ligases. Other DUBs, such as USP14, participate in controlling the UPS through various mechanisms, some of which appear to be mutually antagonistic. USP14 can recycle Ub for reuse when the proteasome interacts with substrates, enhancing proteasome function, and can also preemptively ubiquitinate substrates to prevent their degradation when they are not bound to the proteasome. This highlights the significant role of DUBs in stabilizing the UPS and the overall function of the nervous system.^10^ We emphasize the balancing role of DUBs in the nervous system. Additionally, due to the specificity of cells and substrates, DUBs may also be potential therapeutic targets for certain neuroinflammatory or degenerative conditions, which is promising for the treatment of neurodegenerative diseases.

### 5.3 Ubiquitination defects in metabolic disorders

Metabolic syndrome, maybe defined differently across various studies, generally refers to excessive accumulation of lipids in visceral fat tissue, accompanied by hypertension, hyperglycemia, or hyperlipidemia.^266^, ^267^ Metabolic syndrome is currently estimated to affect over a billion people worldwide, significantly increasing the risks of cardiovascular disease and stroke, two of the leading causes of mortality.^268^ Factors such as inflammation, excessive oxidative stress, and more specifically, various key cytokines, protein kinases, and modifications of critical proteins are considered major disruptors of metabolic activities.^268^, ^269^, ^270^ Consequently, the UPS, a key player in protein quality control, is recognized as having a pivotal influence on metabolic diseases. The impact of the UPS on metabolic disorders is multifaceted, affecting various cellular and organismal functions. On a microscopic level, the UPS regulates fundamental protein degradation processes, thereby influencing signal transduction, transcriptional regulation, protein interactions, and even DNA damage repair—all of which are intimately related to metabolic disturbances, including glucose and lipid metabolism. On a macroscopic level, due to its significant impact on basic cellular physiological functions, UPS‐induced disruptions in molecular metabolic pathways can lead to various acute and chronic metabolic diseases, including diabetes, obesity, and related complications.^271^, ^272^ The regulatory role of the UPS on proteins is undeniable; however, it also modulates glucose and lipid metabolism by controlling the levels and activities of key enzymes or proteins. For instance, in obesity and diabetes, adipocyte differentiation is a crucial process in lipid metabolism involving several ubiquitination‐related proteins. C/EBPβ and PPARγ are key regulatory factors whose ubiquitination status directly affects adipocyte differentiation. Excessive ubiquitination leads to their degradation, severely impacting fat formation. Activating transcription factor 4 (ATF4) is also essential for promoting lipogenesis, and the overexpression of USP7 can restore ATF4 deficiency‐induced lipogenesis impairment and participate in ATF4‐induced adipocyte differentiation.^273^ Additionally, DUBs such as USP2, USP7, USP15, USP19, and USP20 have been proven to be involved in lipid metabolism and fat formation, while USP53 may have beneficial effects on obesity control.^272^ In summary, the regulation of key enzymes and proteins associated with the UPS is crucial for controlling lipid metabolism and obesity, making the UPS is a significant therapeutic target for obesity and lipid metabolic disorders. Another focus of metabolic diseases is diabetes, a condition involving carbohydrate metabolism disorders, typically classified into Type 1 diabetes mellitus (T1DM) and Type 2 diabetes mellitus (T2DM).^274^ T1DM primarily results from autoimmune destruction of pancreatic β‐cells, leading to decreased circulating insulin levels. Although T1DM is more akin to a metabolism disorder caused by autoimmune disease, studies suggest that UPS‐associated key enzymes and proteins might be potential therapeutic targets. For instance, USP22^275^, ^276^ functions by inhibiting autoimmunity, while USP18^277^ may alleviate T1DM stimuli by inhibiting type I interferon signaling or countering certain viral actions. T2DM demonstrates a stronger association with the UPS, as disturbances in blood glucose, inflammatory factors, and various lipid metabolites can lead to β‐cell dysfunction and insulin resistance.^278^ Additionally, insulin signaling is a critical factor affecting T2DM, involving several ubiquitination‐related proteins. In T2DM patients, the ubiquitination status of insulin receptor substrates (IRSs) directly affects signaling efficiency. E3 ligases such as WWP1 promote IRS degradation via ubiquitination, weakening insulin signaling and raising blood glucose levels.^279^ Furthermore, many DUBs have been shown to participate in insulin signaling across various tissues, including USP1 (in pancreatic B‐cells), USP2 (in the liver), USP19 (in white adipose tissue), and USP20 (in skeletal muscle).^272^ In conclusion, given the pivotal role of ubiquitination in metabolic diseases, developing targeted therapeutic strategies is of significant clinical importance. Future research should delve deeper into the specific mechanisms of ubiquitination in metabolic diseases and focus on developing targeted drugs to achieve effective treatment and prevention of metabolic diseases.

### 5.4 Implications in infectious diseases

The UPS plays a crucial role in the host immune response to pathogens. However, certain pathogens, such as viruses and bacteria, exploit this system for their own advantage during host invasion. The UPS is involved in nearly every aspect of cellular physiology, and during viral infections, viruses manipulate the host's ubiquitination system to facilitate their own replication and spread. Additionally, viral proteins themselves can be ubiquitinated, with some viruses even encoding their own ubiquitination machinery and DUBs. Normally, host cells utilize their UPS to activate immune response pathways and inhibit viral protein synthesis, thereby combating viral infections.^280^ However, viruses can evade host defenses by interfering with the ubiquitination and degradation of host surface receptors, thus preventing T‐cell recognition.^281^ The exploitation of the UPS by viruses extends beyond these mechanisms. Some viruses, such as human papillomavirus, utilize E3 Ub ligase complexes to degrade cell cycle regulatory proteins. Moreover, viruses can interfere with the host immune response by using the UPS to avoid detection by the immune system while enhancing their own replication. Overall, dysregulation of the ubiquitination pathway can severely impair the host's ability to defend against viral infections. Similar to viral infections, the host has specific signaling pathways to respond to bacterial infections. However, bacteria can interfere with or hijack these pathways, inhibiting the activation of immune responses. To successfully invade the host, these pathogenic microorganisms often disrupt relevant signaling pathways to evade immune detection, relying heavily on their manipulation of the host's UPS. Bacterial pathogens have evolved to mimic host ubiquitination enzymes, including various E3 ligases such as RING, HECT, and novel E3 ligases.^282^ Furthermore, due to the reversible nature of ubiquitination, bacteria often produce DUBs that mimic host enzymes, thereby protecting substrate proteins from degradation.^283^ In summary, pathogenic infections demonstrate how microorganisms exploit the UPS to manipulate and evade host immune responses, thereby supporting their replication and growth. In infectious diseases, the UPS is extensively involved in pathogen replication, spread, and host immune responses. The interaction between pathogens and the host, as well as the disruption and regulation of immune responses, may provide significant insights for future research in anti‐infective therapies. Therefore, greater emphasis should be placed on the role of the UPS in infectious disease control, as it may present opportunities for novel anti‐infective treatments or vaccine development.

## 6 THERAPEUTIC TARGETING OF UBIQUITINATION

Ubiquitination functions as a critical regulatory mechanism for maintaining protein quality and quantity across diverse cell types, offering potential avenues for developing specific modulators to either augment or inhibit this process. Precise control of ubiquitination, minimizing detrimental side effects, could signify a transformative breakthrough in influencing human health outcomes. Nonetheless, the intrinsic complexity of proteins and the Ub system presents substantial challenges, rendering this objective largely unattained. At present, the identification of inhibitors targeting ubiquitination has been limited to small molecules, with notable examples including bortezomib and carfilzomib, which are already utilized in clinical oncology. In the context of neurodegenerative diseases, an alternative therapeutic strategy involves exploiting the reversible nature of ubiquitination through the application of DUBs to restore cellular homeostasis. Additionally, a burgeoning area of research in this domain is the development of proteolysis‐targeting chimeras (PROTACs), which facilitate the targeted degradation of specific proteins via recruiting them to E3 ligases. Nevertheless, this system is still under development and investigation, and effective regulation of ubiquitination remains a work in progress. Here, we summarize the drugs and enzymes targeting ubiquitination across various diseases, as outlined in Table 2.

**TABLE 2:** The role and function of DUBs in related diseases.

Disease name	DUBs	The role and function of DUBs	References
Cancer	USP3, USP4, USP5, USP7, USP11, USP14, USP17, USP27X, USP29, USP36, USP37, USP42, USP46, USP47, A20, CSN5, OTUB1, OTUD4, UCHL1	Upregulation of epithelial–mesenchymal transition in cancer	^199^, ^284^
	USP2A, USP3, USP4, USP6, USP13, USP15, USP16, USP17, USP21, USP22, USP27X, USP28, USP38, USP39, USP42, USP43, USP49, USP51, CSN5, POH1, TRABID, UCHL1, UCHL3, BRCC36, COPS6, COPS5, ATXN3L, ATXN3, OTUD7B, OTUD2	Upregulation of tumor invasion in cancer	^284^, ^285^, ^286^, ^287^, ^288^, ^289^, ^290^, ^291^, ^292^, ^293^, ^294^, ^295^, ^296^, ^297^, ^298^, ^299^
	USP9X, USP53, OTUD3	Downregulation of tumor invasion in cancer	^199^, ^284^, ^300^, ^301^
	USP3, USP4, USP8, USP9X, USP17, USP22, USP27X, USP29, USP37, USP45, UCHL1	Upregulation of tumor migration in cancer	^199^, ^284^
	USP11, USP53	Downregulation of tumor migration in cancer	^199^, ^284^
	USP1, USP4, USP7, USP10, USP11, USP20, USP21, USP24, USP26, USP33, A20, ataxin 3, CSN5, OTUB2, UCHL1, AMSH, COPS6, COPS5, UCHL5	Upregulation of metastatic dissemination in cancer	^199^, ^217^, ^284^, ^298^, ^299^, ^300^, ^301^, ^302^, ^303^, ^304^, ^305^, ^306^, ^307^
	OTUD1, TRABID, OTUD7A, OTUD6B, CYLD	Downregulation of metastatic dissemination in cancer	^199^, ^284^, ^308^, ^309^, ^310^, ^311^, ^312^, ^313^, ^314^, ^315^, ^316^
Neurodegenerative diseases and other nervous system disorders	ATXN3, USP7, USP8, USP14, USP15, USP30, BAP1, CYLD, PSMD14, TNFAIP3, UCH‐L1, UCHL‐5, USP1, USP4, USP10, USP11, USP12, USP13, USP18, USP22, USP33, USP36, USPL1, USP9X, YOD1	Involved in the upregulation of neurodegeneration formation	^61^, ^243^, ^317^, ^318^, ^319^, ^320^, ^321^, ^322^, ^323^, ^324^, ^325^, ^326^, ^327^, ^328^, ^329^, ^330^, ^331^, ^332^, ^333^, ^334^, ^335^, ^336^, ^337^, ^338^, ^339^, ^340^
	USP16, A20, CYLD, OTULIN, USP18, USP25, UCHL1, UCHL3, UCHL5/UCH37, USP2, USP5, OTUD4, PSMD14, EIF3H, AMSH	Involved in the progression of other nervous system disorders	^10^, ^341^, ^342^, ^343^, ^344^, ^345^, ^346^, ^347^, ^348^, ^349^
Obesity	USP2, USP7, USP15, USP19, USP20	Involved in the progression of worsening obesity	^271^, ^350^, ^351^, ^352^
	USP53	Involved in the progression of reducing obesity	^271^, ^353^
T1DM	USP18, USP22	Involved in the progression of worsening T1DM	^275^, ^277^, ^350^
T2DM	USP1, USP19, USP21, USP2, USP14, USP20	Involved in the progression of worsening T2DM	^354^, ^355^, ^356^, ^357^, ^358^
	USP22, USP2, USP9X, USP20, USP33, USP4, USP7, USP10, USP18	Involved in the progression of reducing T2DM	^356^, ^359^, ^360^, ^361^, ^362^, ^363^, ^364^, ^365^, ^366^

### 6.1 Small molecule inhibitors and activators of ubiquitination enzymes

The ubiquitination pathway plays a crucial role in intracellular protein degradation and signal transduction, with its dysregulation closely linked to the onset and progression of various diseases, particularly cancer and neurodegenerative disorders. Therefore, developing small molecule drugs targeting specific enzymes within the ubiquitination pathway, such as E1, E2, and E3 enzymes, as well as proteasome inhibitors or activators, holds significant therapeutic potential.^14^ In the field of cancer, recent research has focused on small molecule inhibitors targeting E3 ligases. These inhibitors can specifically prevent the ubiquitination and degradation of tumor‐associated proteins, thereby affecting tumor growth and spread.^47^ Additionally, PROTACs represent an emerging therapeutic strategy. PROTACs work by linking E3 ligases to target proteins (proteins of interest, POIs), inducing the ubiquitination and subsequent degradation of the POIs. Due to their high selectivity and specificity, PROTACs offer a novel direction for cancer treatment.^367^, ^368^ Furthermore, activating Ub ligases is also a therapeutic strategy for addressing abnormal protein accumulation in neurodegenerative and metabolic diseases. For example, NRBP1‐containing CRL2/CRL4A complexes can target and promote the degradation of Aβ protein in AD, enhancing related ubiquitination pathways and slowing disease progression.^369^ While the development of agonists for Ub ligases is still in its early stages, research on small molecule inhibitors targeting DUBs is more advanced and extensive, with significant therapeutic potential. DUBs can remove Ub tags from proteins, affecting their stability and function and playing a role in protein quality control. In certain disease states, such as cancer and neurodegenerative diseases, overactivation of DUBs may lead to the accumulation of abnormal proteins.^256^ Developing small molecule inhibitors for DUBs can inhibit their activity, enhance the ubiquitination process, and promote the degradation of abnormally accumulated proteins, thereby effectively inhibiting disease progression. For example, small molecule inhibitors targeting USP7 can stabilize p53 and inhibit cancer progression.^369^ Additionally, USP7 inhibitors can potentially target neurological diseases by inhibiting neuroinflammation.^370^ Other examples include inhibitors targeting USP14, which have been widely studied for the treatment of neurodegenerative diseases.^243^ In summary, developing small molecule drugs targeting specific enzymes within the UPS, such as inhibitors or activators of E1, E2, or E3 enzymes, as well as DUB inhibitors, holds significant potential for disease treatment. These drugs can influence the ubiquitination process of specific substrates, aiding in disease control and providing new strategies for treating various diseases. Future research should focus more on the specific mechanisms of ubiquitination in related diseases, especially the key molecular mechanisms underlying abnormal proteins or disease progression, to achieve effective treatment and prevention. Next, we will discuss the potential of targeting the UPS in cancer and neurodegenerative disease therapies.

### 6.2 Targeting ubiquitination for cancer therapy

Approximately 80−90% of intracellular proteins are removed through the UPS. Tumor cells often exploit the UPS to reduce apoptosis or increase proliferation. Consequently, targeting the ubiquitination or proteasome system with various drugs has become a viable strategy for cancer treatment. Increasing recognition of the UPS as a target for cancer therapy has stimulated the development of small molecules, peptides, and proteasome inhibitors that can restore cellular balance and disrupt tumor growth.^371^ These targeted therapies can be classified based on their targets: E1 enzymes (e.g., MLN7243 and MLN4924), E2 enzymes (e.g., leucettamol A and CC0651), E3 ligases (e.g., nutlin and MI‐219), proteasome inhibitors (e.g., bortezomib, carfilzomib, oprozomib, and ixazomib), and DUB inhibitors (e.g., G5 and F6).^14^ Many of these targeted drugs have achieved tangible success in cancer treatment, offering new hope. For instance, proteasome inhibitors like Bortezomib or MG132 block the degradation of proteins entirely. However, while effective, such drugs may lack specificity and selectivity, suggesting that drugs targeting specific E3 ligases might offer better selectivity and lower toxicity, fulfilling clinical needs more effectively.^47^ Among various targeted small molecule inhibitors, those targeting E3 ligases are the most extensively researched.^215^

E3 ligases play a pivotal role in tumorigenesis by promoting protein ubiquitination and degradation, thereby influencing various physiological processes.^372^, ^373^ A notable example is the RING‐type E3 ligase MDM2, which is overexpressed in many human cancers.^374^ MDM2 directly interacts with p53, a critical gene involved in cell cycle arrest, DNA repair, and apoptosis regulation, making it a significant cancer therapy target.^375^ MDM2 induces the ubiquitination and proteasomal degradation of p53, reducing its tumor suppressor functions. Consequently, numerous small molecule inhibitors targeting MDM2 have been designed.^376^ Nutlin, a cis‐imidazoline analog, was among the first inhibitors identified to bind MDM2, preventing p53 degradation. Similarly, MI‐219 disrupts the MDM2–p53 interaction.^377^ Unlike the aforementioned inhibitors, RITA (Reactivation of p53 and Induction of Tumor cell Apoptosis) directly binds to p53, blocking its interaction with ^MDM2^ and potentially other similar proteins, thereby promoting p53 activation in tumors.^377^, ^378^ The second‐generation MDM2 inhibitor, RG7388 (idasanutlin), has demonstrated reduced toxicity and increased efficacy compared to early Nutlins.^379^ Another promising E3 ligase target within the SCF (Skp1–cullin–F‐box) complex is the F‐box protein Skp2, which is overexpressed in various cancers and involved in tumor regulation.^380^ Skp2 inhibitors like SKPin C1 and SMIP004 have shown tumor‐inhibitory effects.^381^

Compared to traditional small molecule inhibitors, PROTACs offer a practical approach to protein degradation. By recruiting E3 ligases to POIs, PROTACs induce the ubiquitination and proteasomal degradation of the POIs. Commonly targeted E3 ligases for PROTACs include MDM2, IAPs, cereblon, and VHL.^382^ Several PROTAC‐based inhibitors, such as ARV‐471 and ARV‐110, have entered clinical trials.^367^ Numerous PROTACs have been successfully developed, with many undergoing clinical validation for cancer treatment.^368^ Overall, drugs targeting E3 ligases represent an effective strategy among UPS‐targeted therapies, with PROTAC technology offering superior selectivity and potency. While other UPS targets, such as E1 and E2 enzymes, are also being actively researched and tested, each has its own advantages and limitations. Finding and developing more efficient targets and drugs remain crucial for advancing cancer treatment.

### 6.3 Potential for drug development in neurodegenerative diseases

Proteasome dysfunction is observed in AD, PD, HD, and ALS, a common feature across nearly all neurodegenerative diseases. These conditions often involve the accumulation of abnormal proteins, suggesting an insufficient proteasomal clearance capability. Thus, enhancing ubiquitination or inhibiting deubiquitination processes through targeting the UPS may be an optimal approach for treating neurodegenerative diseases. One strategy is to promote the degradation of abnormal proteins. For instance, in AD, the aggregation of Aβ is a key factor in disease progression. Drugs can be designed to activate E3 ligases, such as NRBP1‐containing CRL2/CRL4A complexes, to accelerate Aβ degradation by targeting regulators like BRI2 and BRI3.^369^ In PD, developing drugs that activate E3 ligases like CHIP could promote the ubiquitination and degradation of α‐synuclein.^383^ Another strategy involves inhibiting DUB, making them potential therapeutic targets for neurodegeneration.^265^ While the structure and characteristics of many DUBs have been studied, their precise physiological functions remain largely unknown. However, growing research highlights their potential role and core involvement in treating neurodegenerative diseases.^265^ The integrity of neural development is closely linked to specific DUBs and their associated ubiquitination pathways, with a balance between ubiquitination and deubiquitination essential for maintaining neural stability. Neurodegenerative diseases frequently involve neuronal death, mitochondrial dysfunction, ER stress, and inflammation, with ubiquitination and deubiquitination playing significant roles in these processes. Additionally, UPS regulation can affect synaptic function, with synaptic loss being a critical cause of cognitive and motor deficits in many neurodegenerative diseases. Ubiquitination is crucial for the renewal and maintenance of synaptic proteins. Thus, developing drugs to modulate the ubiquitination state of synaptic‐related proteins may help restore synaptic function and improve cognitive and motor abilities. Specific DUBs known to function at synapses include UCHL1 (involved in maintaining synaptic function and structure), USP14 (enhancing proteasome function by recycling Ub in the synaptic proteasome), Fat Facets (Faf), and USP9X (both involved in cell differentiation and synaptic function).^265^ Beyond promoting abnormal protein degradation, protecting neuronal survival and function, and improving synaptic function, DUB inhibitors might offer significant advantages due to their substrate specificity, making them potential therapeutic targets in the Ub–proteasome pathway. Similar to cancer treatment, drugs directly targeting the proteasome have shown success but often cause unintended side effects due to a lack of specificity. High‐throughput screening has identified several small molecule inhibitors, including those targeting USP7 and UCHL1.^384^

## 7 FUTURE PERSPECTIVES AND CHALLENGES

### 7.1 Emerging roles of ubiquitination in biology and medicine

The discovery of canonical ubiquitination dates back several decades, but its significance extends far beyond the classical understanding of ubiquitination process. Initially, ubiquitination was thought to be confined to proteins. However, recent research has revealed the diversity of ubiquitination substrates, encompassing nucleotides, sugars, and lipids. These substrates perform various functions postubiquitination, such as glycogen clearance and bacterial elimination. Additionally, the chemical bonds between the Ub complex and its substrates include ester and amide bonds, in addition to the conventional peptide and isopeptide bonds^385^, ^386^, ^387^ Consequently, the concept of noncanonical ubiquitination has been proposed. One aspect of noncanonical ubiquitination involves the variation in Ub linkage types. Studies indicate that, besides lysine, amino acids like serine (S), threonine (T), and cysteine (C) can serve as alternative Ub linkage sites. Notably, S and T residues form ester bonds during ubiquitination, although these bonds are less stable than peptide bonds. Despite its potential, the abundance and kinetics of nonpeptide‐linked ubiquitination remain underexplored, marking a promising avenue for future research.^21^, ^388^

In addition to variations in canonical ubiquitination processes, researchers have identified Ub‐like molecules that perform functions analogous to ubiquitination, yet with distinct cellular roles. One of the extensively studied examples is the small Ub‐like modifier (SUMO). The SUMO system, while simpler in composition than the ubiquitination system, modifies a broader range of substrates. Similar to ubiquitination, SUMOylation can alter protein localization, activity, and stability.^389^ At the microscopic level, the SUMO system participates in various cellular functions and processes. SUMO modifies functionally related protein groups to primarily regulate nuclear processes, including gene expression, DNA damage response, RNA processing, cell cycle progression, and protein deposition. Current research has expanded SUMO's role to include immunoregulation, cell migration, and pathophysiological processes.^390^, ^391^ On a broader scale, SUMOylation is implicated in the pathogenesis of multiple diseases. For example, due to its role in stress response, SUMO is upregulated in many types of cancer cells, serving as a mechanism to protect these cells.^392^ The relationship between SUMOylation and ubiquitination extends beyond mere similarity. SUMO can integrate within the Ub modification system, forming Ub–SUMO hybrid polymers. The ubiquitination of SUMO can induce proteasomal degradation of substrates.^24^ Analogous to SUMO in its function, ISG15 is a 15‐kDa Ub‐like protein composed of two Ub‐like domains connected by a short linker. The corresponding process, known as ISGylation, involves a three‐enzyme cascade. Similar to ubiquitination, ISGylation is reversible. Linked with interferon responses, ISG15 plays a crucial role in antiviral defenses.^393^ Additionally, another well‐known Ub‐like modifier is NEDD8, which has become a significant target in the development of anticancer therapeutics.^394^

### 7.2 Technological advancements and tools for studying ubiquitination

The tools for investigating ubiquitination have become increasingly diverse. For example, super‐resolution microscopy enables the observation of subcellular structural details of ubiquitination events, such as the subcellular localization of the 19S and 20S proteasome complexes. This technique has also revealed the nuclear localization of the E3 Ub ligase RAD18 during its participation in membrane‐associated DNA repair.^395^ Furthermore, live‐cell imaging allows for real‐time tracking of the ubiquitination pathways within cells. It has been used to observe the recruitment of OPTN to PARK2‐mediated ubiquitinated mitochondria.^396^ Technologies such as live‐cell imaging and mass spectrometry are highly convenient for mechanistic studies of ubiquitination.^397^

Molecular dynamics simulations can further elucidate the molecular mechanisms involved in ubiquitination and assist in studying the structure‐function relationships of ubiquitination regulatory factors. For instance, it has been validated that ubiquitination within the reticulon homology domain of the ER‐phagy receptor FAM134B promotes receptor aggregation and its binding with lipidated LC3B, thus stimulating ER autophagy.^398^

With the evolution of artificial intelligence (AI), its application in ubiquitination research has become increasingly significant. Current machine learning models can predict binding behaviors among Ub, E3 ligases, and substrate ligands.^399^ Moreover, a network system named DEGRONOPEDIA has been developed; it searches for degrons, maps them onto adjacent residues that can undergo ubiquitination and disordered regions, and assesses N‐/C‐terminal stability.^285^ The application of AI in ubiquitination research is not limited to classical ubiquitination. For instance, GPS‐SUMO is a predictor constructed using three computational algorithms (deep neural networks, penalized logistic regression, and transformers), allowing users to accurately predict SUMO sites within a cell by inputting one or multiple protein sequences or identifiers, and it has been made freely available by its developers.^286^ Furthermore, the PROTAC system for targeted protein degradation has also been integrated with a deep neural network model, resulting in the development of a system called DeepPROTACs.^287^ AI is still in its infancy regarding the precise localization of protein molecules and the functionality optimization of targeted degradation, offering substantial room for research and advancement.

### 7.3 Challenges in targeting ubiquitination for therapeutic interventions

Modulating the ubiquitination process to regulate disease progression remains a significant challenge in current research. Although several ubiquitination inhibitors have received United States Food and Drug Administration (US FDA) approval, no ubiquitination agonists have been approved to date. For instance, ixazomib, an oral proteasome inhibitor, has been used in the treatment of multiple myeloma.^288^ Earlier US FDA‐approved ubiquitination inhibitors include Bortezomib and Carfilzomib, which are also used for treating multiple myeloma.^289^, ^290^ These targeted drugs are part of the broader strategy of targeted protein degradation. However, due to the diversity of Ub enzymes and substrates, and the specificity required for targeted therapies, developing universally applicable targeted drugs is quite challenging. In this context, drug research has also focused on improving drug delivery systems, such as enhancing the efficacy of Bortezomib in treating hepatocellular carcinoma through nanosystem‐mediated delivery.^291^ Despite these advances, more efficient and safer technologies are still under development. Additionally, the reversible nature of the ubiquitination process presents complex challenges for therapeutic interventions. Understanding the dynamics of E3 ligases and DUBs is crucial for studying the broader dynamics of ubiquitination. In summary, research on ubiquitination is still in its early stages. Addressing issues of specificity and universality, integrating advanced drug delivery technologies, and undergoing extensive basic and clinical trials are essential steps for the maturation of ubiquitination research.

## 8 CONCLUSION

### 8.1 Summary of key findings

The discovery of ubiquitination has expanded our understanding of protein quality control mechanisms beyond the lysosomal pathway. Subsequent findings revealed that ubiquitinated proteins can be targeted to both the proteasome and the autophagosome, thereby adding more possibilities to the process of autophagy. Ubiquitination is a three‐enzyme cascade reaction involving E1, E2, and E3 ligases. The wide variety of these enzymes contributes to the specificity and diversity of the ubiquitination process. DUBs, acting as Ub recyclers, render ubiquitination reversible. Originally, it was believed that ubiquitination was exclusive to proteins. However, it has been proven that sugars and lipids can also be ubiquitinated. The sites on substrates where Ub attaches are known as UBDs. Based on the discovery of UIM and UBA, UBDs exist in various topologies within substrates, guiding Ub attachment and subsequent action. Ub receptors present in the proteasome recognize ubiquitinated proteins, which are then degraded into short peptides after Ub binds to these receptors. Ub is a ubiquitous molecule in living organisms, and its widespread distribution underpins its complex and diverse functions. The most well‐known function of ubiquitination is protein degradation. Misfolded proteins from various cellular locations are directed to the proteasome for degradation, facilitated by conformational changes in the proteasome. In protein localization and transport, especially for secretory proteins, ubiquitination plays a critical role in sorting and signaling across different organelles. For instance, in ERAD pathway, misfolded proteins are recognized, relocalized, and subsequently degraded by the proteasome into short peptides, which are then transported to the cytoplasm or nucleus as needed. Ubiquitination can also influence vesicle size and assembly. In the downstream processes of protein secretion, ubiquitination acts as a signal for proteins to enter and exit the Golgi apparatus. Nuclear import of proteins is also regulated by ubiquitination, underscoring its importance in altering protein localization and various cellular functions. In numerous signaling pathways, ubiquitination targets specific key proteins, thereby altering the direction and outcome of these pathways. The structural and functional attributes of ubiquitination make it crucial for maintaining biological homeostasis. When functioning properly, ubiquitination helps maintain protein homeostasis, nucleic acid stability, cellular proliferation, and immune balance. Conversely, its dysregulation can lead to a range of diseases, including tumors, neurodegenerative disorders, metabolic conditions, and infectious diseases. Understanding the mechanisms of these diseases and targeting the ubiquitination process—either by inhibiting or activating it—presents a promising therapeutic approach.

### 8.2 Implications for understanding disease mechanisms and therapeutic development

Ubiquitination is a complex process that involves the attachment of Ub molecules to target proteins via various Ub enzymes, followed by the interaction between these enzymes and substrate molecules, and ultimately the transport of these substrates to the proteasome for degradation. Each step in this process presents potential therapeutic targets for disease treatment. Currently identified therapeutic targets within the ubiquitination pathway include: (1) the reversible nature of the UPS; (2) targeting various enzymes involved in ubiquitination to block the process; (3) development of modulators specific to E3 ligases; (4) she emerging PROTACs system. Although various molecules have been identified in existing studies, the progress in treating specific diseases remains unclear. Continued investigation into the molecular basis of diseases caused by UPS dysregulation and the development of targeted therapeutics is crucial for advancing disease treatment and prevention. With the rapid development of other disciplines, the integration of AI technology and materials science with ubiquitination‐targeted drugs or strategies can potentially enhance the efficiency of diagnostic and therapeutic methods.

### 8.3 Future directions for research in ubiquitination biology

Ubiquitination is an “ancient” molecular process, earning its name “ubiquitin” due to its ubiquitous presence across cellular life. It is indispensable in cellular processes, with its dysregulation leading to various pathological conditions and diseases. However, the number of drugs targeting ubiquitination, especially those approved by the US FDA, remains scarce. Notably, there are currently no activators of the ubiquitination process available, posing a challenge for treating diseases caused by suppressed ubiquitination. Given the advancement of emerging technologies, researchers are now exploring the integration of ubiquitination with nanotechnology, AI, and other cutting‐edge technologies, aiming to develop more precise and intelligent diagnostic and therapeutic techniques targeting ubiquitination. Nonetheless, continued research in the field of ubiquitination is essential. The molecular diversity of ubiquitination and its various forms inherently pose challenges in achieving specificity. Furthermore, given the widespread distribution of Ub and its substrates (proteins, sugars, lipids), there remains substantial scope for research. The ongoing decryption of ubiquitination processes should persist to unlock its full potential and therapeutic applications.

## AUTHOR CONTRIBUTIONS

*Conceptualization and writing—original draft*: Yan Liao. *Writing—review*: Wangzheqi Zhang. *Supervision*: Yang Liu. *Conceptualization and writing—review*: Chenglong Zhu. *Supervision and funding acquisition*: Zui Zou. All authors have read and approved the final manuscript.

## CONFLICT OF INTEREST STATEMENT

The authors declare that they have no conflict of interest.

## ETHICS STATEMENT

Not applicable.

## Data Availability

All data are freely available from the corresponding author upon request.
